# Heterologous expression of a thermophilic diacylglycerol acyltransferase triggers triglyceride accumulation in *Escherichia coli*

**DOI:** 10.1371/journal.pone.0176520

**Published:** 2017-04-27

**Authors:** Beatriz Lázaro, Juan A. Villa, Omar Santín, Matilde Cabezas, Cintia D. F. Milagre, Fernando de la Cruz, Gabriel Moncalián

**Affiliations:** 1 Departamento de Biología Molecular, Universidad de Cantabria and Instituto de Biomedicina y Biotecnología de Cantabria (IBBTEC), Consejo Superior de Investigaciones Científicas - Universidad de Cantabria, C/ Albert Einstein, Santander, Cantabria, Spain; 2 Department of Organic Chemistry, Institute of Chemistry, Universidade Estadual Paulista (UNESP), Rua Prof. Francisco Degni, Araraquara, São Paulo, Brazil; Karl-Franzens-Universitat Graz, AUSTRIA

## Abstract

Triglycerides (TAGs), the major storage molecules of metabolic energy and source of fatty acids, are produced as single cell oil by some oleogenic microorganisms. However, these microorganisms require strict culture conditions, show low carbon source flexibilities, lack efficient genetic modification tools and in some cases pose safety concerns. TAGs have essential applications such as behaving as a source for added-value fatty acids or giving rise to the production of biodiesel. Hence, new alternative methods are urgently required for obtaining these oils. In this work we describe TAG accumulation in the industrially appropriate microorganism *Escherichia coli* expressing the heterologous enzyme tDGAT, a wax ester synthase/triacylglycerol:acylCoA acyltranferase (WS/DGAT). With this purpose, we introduce a codon-optimized gene from the thermophilic actinomycete *Thermomonospora curvata* coding for a WS/DGAT into different *E*. *coli* strains, describe the metabolic effects associated to the expression of this protein and evaluate neutral lipid accumulation. We observe a direct relation between the expression of this WS/DGAT and TAG production within a wide range of culture conditions. More than 30% TAGs were detected within the bacterial neutral lipids in 90 minutes after induction. TAGs were observed to be associated with the hydrophobic enzyme while forming round intracytoplasmic bodies, which could represent a bottleneck for lipid accumulation in *E*. *coli*. We detected an increase of almost 3-fold in the monounsaturated fatty acids (MUFA) occurring in the recombinant strains. These MUFA were predominant in the accumulated TAGs achieving 46% of the TAG fatty acids. These results set the basis for further research on the achievement of a suitable method towards the sustainable production of these neutral lipids.

## Introduction

Triglycerides (TAGs), water-insoluble fatty acid triesters of glycerol, constitute the main components of lipid inclusion bodies in living organisms being the major storage molecules of metabolic energy and source of fatty acids (FA). Regulation of cellular membrane fluidity by preventing certain FAs from entering phospholipid biosynthesis or deposit of reducing equivalents have been also discussed as key functions of TAGs and the lipophilic bodies they form in the cytoplasm [1–4]. The occurrence of intracellular TAG reservoirs is widespread in many eukaryotes, while its accumulation in prokaryotes is restricted only to a few aerobic heterotrophic bacteria and some cyanobacteria. Biosynthesis of TAGs is a common property of species belonging to the Gram-positive actinomycetes group (such as *Mycobacterium* sp., *Nocardia* sp., *Rhodococcus* sp., *Micromonospora* sp., *Dietzia* sp., *Gordonia* sp. and streptomycetes). These bacteria might be defined as oleaginous organisms since they accumulate more than 20% of their biomass as lipids [1]. TAG inclusions together with wax esters (WEs) have been also reported for some Gram-negative proteobacteria like *Alcanivorax* sp., *Marinobacter* sp. or *Acinetobacter* sp. [5,6], though the amount of accumulated TAGs is smaller in these species. In bacteria the final step in WEs and TAGs biosynthesis is catalyzed by a soluble amphiphilic enzyme that is only attached to the membrane by electrostatic interactions [7]. This family of promiscuous enzymes, the wax ester synthase/acyl-CoA:diacylglycerol acyltransferases (WS/DGAT), mediates both WE and TAG formation from long-chain acyl-coenzyme A (CoA) molecules as acyl donors and long-chain fatty alcohols or diacylglycerols (DAGs) as respective acyl acceptors in bacteria. Therefore, formation of TAGs by WS/DGAT depends on the presence of DAGs and acyl-CoA donors in the cell. Sequence analysis of several homologs of the enzyme responsible for the final step of TAG biosynthesis underscored a highly conserved motif HHxxxDG occurring in all members of that family as well as in other bacterial enzyme classes. Moreover, this HHxxxDG motif has been proved to participate in catalysis [7]. Plant WS/DGAT are phylogenetically related, the same as most proteobacterial members of this family, whereas the actinomycete ones are disperse along the phylogenetic tree [8].

TAGs have been obtained from animal and plant oils for the production of commercially significant goods, being widely used in both industrial and healthcare applications. Oleaginous seeds are used to produce biodiesel, a substitute for fossil-based fuel consisting of fatty acid methyl esters (FAMEs) and fatty acid ethyl esters (FAEEs). Obtained by chemical transesterification of TAGs with methanol or ethanol [3], the properties of the biofuel (calorific value, fluidity at low temperatures, oxidative stability and nitrogen oxide emissions) are determined by the nature of the acyl chains (mainly length and saturation degree) forming these TAGs [9,10]. In fact, mono unsaturated fatty acids or MUFA, have been reported to be the best components for biodiesel. Hence, the great demand of new bio-resources for the production of all triglyceride-derived products and, specifically, the need of optimizing a method for producing the optimal FAEEs that could be employed for diesel engines substituting the traditional plant-based oil production model, provides motivation to engineer production from a robust microbial platform [11,12]. However, an economic process for microbial production of TAGs at an industrial level has not been established yet. Technical issues for industrial culturing of bacterial species naturally capable of synthesizing sufficient yields of these lipids [13,14] encouraged research on the employment of other microorganisms as host for the expression of WS/DGAT for large-scale TAGs production. The feasibility of lipid production in the well-known model microorganism *Escherichia coli* has been studied recently. *E*. *coli* has been successfully engineered to synthesize jojoba oil-like WEs [15], fatty acid butyl esters (FABEs) and FAEEs [3,15] and fatty-acid-derived hydrocarbons [16]. This process has been achieved both in WS/DGAT expressing *E*. *coli* strains with deleted genes [17–20] and in wild-type (WT) *E*. *coli* strains overexpressing additional proteins [12,19,21,22]. However, recombinant oil production through the heterologous expression of a WS/DGAT has not been achieved in WT *E*. *coli* strains without further modification. In the present work a codon-optimized gene coding for a WS/DGAT from a Gram-positive thermophilic bacteria, *Thermomonospora curvata* [23], was transformed into the industrially suitable organism *E*. *coli*. Proving the *in vivo* activity of this thermophilic enzyme (tDGAT), we successfully introduced the triacylglycerol biosynthesis pathway in the gamma-proteobacteria. Furthermore, its activity in an industrially appropriate microorganism, giving rise to the production and accumulation of TAGs, sets the basis for further research on the achievement of a suitable method for sustainable production of these neutral lipids. At the same time, the lipid composition observed led us to think that the WS/DGAT might have possible new specificities, making it highly attractive for biotechnological applications such as biodiesel production.

## Results

### *Thermomonospora curvata* acyltransferase ACY99349 belongs to the WS/DGAT family

Enzymes belonging to the WS/DGAT family are widely distributed. Homologs can be found in prokaryotes, plants, protists and cnidaria, arthropoda or hemichordata animals [24]. On the other hand, highly stable enzymes produced by thermophilic organisms are known to expand the range of reaction conditions suitable for biocatalysis [25,26]. Thus, with the aim of finding a stable WS/DGAT enzyme, NCBI BLAST analysis [27] focused on thermophilic organisms was carried out using the sequence of *Marinobacter hydrocarbonoclasticus* Ma2 [UniProt:A1U572] [28]. This led to the finding of a putative WS/DGAT [Uniprot: ACY99349], named tDGAT hereafter, from *Thermomonospora curvata* DSM 43183. tDGAT was found to be 33% identical to Ma2. *T*. *curvata* DSM 43183 is an aerobic, cellulolytic and thermophilic actinobacteria with an optimal growing temperature of 50°C [23].The full-length putative *tDGAT* gene had 1,143 base pairs and coded for a 482-amino acid polypeptide with a molecular mass of 51.72 kDa and a theoretical isoelectric point (pI) of 6.44.

**Fig 1:**
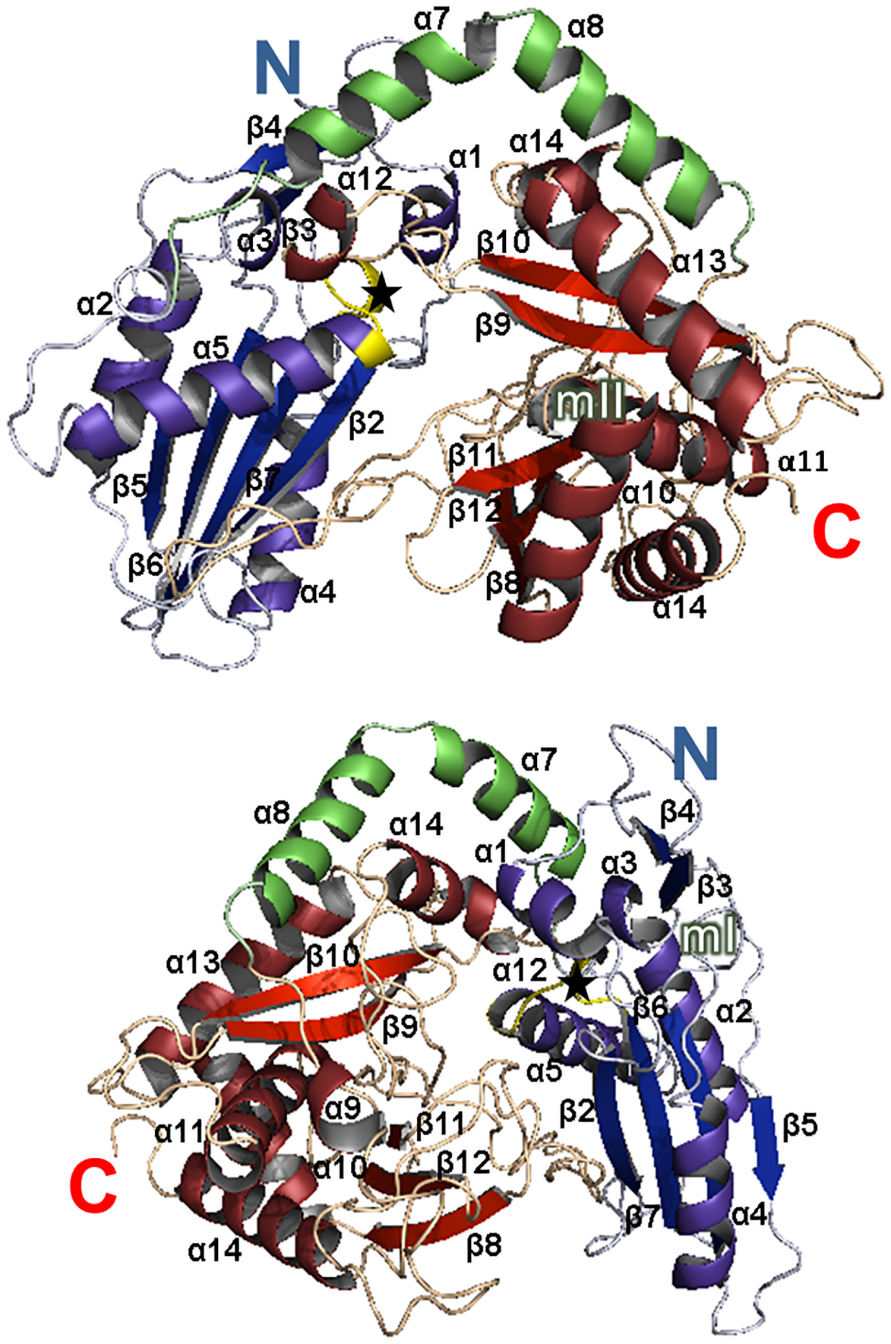
tDGAT belongs to WS/DGAT family. Structural prediction analysis of the tDGAT protein revealed a monomer with a two-domain structure: N-terminal domain is colored in blue, C-terminal domain in red and the connecting loops in green. Beta sheets and alpha helices are shown in darker colors and labeled with the same notation used in the alignment in S1 Fig. The active site HHxxxDG is also marked with a black star and colored in yellow. Other important conserved motifs are labeled (mI and mII).

The three-dimensional (3D) model of the tDGAT protein was predicted through the PHYRE2 Web server [29,30]. Like other members of this family, tDGAT is predicted to have an acyl-CoA-dependent acyltransferase fold, with two domains connected by a helical linker (Fig 1). The core of the predicted N-terminal domain contains a four-stranded mixed sheet (β2, β5, β6 and β7) surrounded by three alpha-helices (α2, α4 and α5). The core of the predicted C-terminal domain consists of a five-stranded mixed sheet (β8, β9, β10, β11 and β12) and five alpha-helices (α9, α10, α11, α13 and α14) covering the external face of the mixed sheet.

The protein sequence of tDGAT was aligned with representative members of the WS/DGAT family (S1 Fig) through the software T-Coffee [31]. tDGAT contains all the conserved motifs characteristic of WS/DGAT [8,32]: mainly the catalytic site ^140^HHaavDG^146^, motif I ^118^PLW^120^and motif II ^281^ND^282^. Like in other acyl-CoA-dependent acyltransferases [33] the catalytic motif ^140^HHaavDG^146^, in the N-terminal domain of tDGAT, is predicted to be located in the hydrophobic pocket or channel that restricts the accessibility of hydrophilic substrates. Motif I is in a connecting loop between the helix α4 and the sheet-strand β6, spatially close to the catalytic center, leading to think it is presumably involved the orientation of the catalytic residues. Motif II appears in one side of the central tunnel, which suggests a crucial role in the interaction with the substrate accessing the catalytic site. In fact, docking of palmitoyl-CoA into the donor pocket of the predicted tDGAT structure showed that the palmitoyl moiety is sandwiched between α5 helix and the β sheet formed by β9 and β10 strands (S2 Fig). Besides, multiple sequence alignment (S1 Fig) shows a mismatch in the residues corresponding to the α7 and α8 helices of the predicted 3D structure of tDGAT. These helices connect the N- and C-terminal domains of the enzyme. The amino acids in the protein region corresponding to the helical linker are poorly conserved among the WS/DGAT members, being longer in tDGAT than in the other representative members of the family.

The theoretical pI and the GRAVY (Grand average of hydropathicity) of all the aligned proteins were calculated through the Protparam tool of Expasy (www.expasy.org). Table 1 shows that the only protein among the most representative WS/DGAT giving rise to a positive GRAVY, meaning it is the most hydrophobic protein, was tDGAT. In addition, according to the theoretical isoelectric point (pI) this thermophilic protein would be negatively charged in an environment with a neutral pH.

**Table 1:** Representative proteins of the WS/DGAT family.

Protein name	Organism	Database accession number	Size(amino acids)	Hydrophobic amino acids (AFLIVPG)	Polar amino acids (QNHSTYCMW)	Negatively charged residues (ED)	Positively charged residues (RK)	Theoretical pI	GRAVY value
tDGAT	***Thermomonospora curvata* DSM 43183**	[NCBI: WP_012854133.1]	482	289	86	55	52	6.44	0.043
Ma2	*Marinobacter aquaeolei* VT8	[NCBI: YP_960328]	473	223	148	48	54	8.97	-0.285
Av1	*Alcanivorax* sp. 97CO-5	[GenBank: EUC69427.1]	451	215	141	44	51	9.12	-0.194
Ma1	*Marinobacter aquaeolei* VT8	[NCBI: YP_957462]	455	207	148	53	47	6.32	-0.295
Ac1	*Acinetobacter* sp. ADP1	[NCBI: YP_045555]	458	213	142	48	55	9.05	-0.203
Ps1	*Psychrobacter cryohalolentis* K5	[NCBI: YP_579515]	479	222	146	51	60	9.23	-0.187
Rh1	***Rhodococcus jostii* RHA1**	[NCBI: YP_701572]	420	214	124	45	37	5.85	-0.126
Ms1	***Mycobacterium* sp**.	[NCBI: YP_001073143]	454	238	120	51	45	6.08	-0.108
Nf1	***Nocardia farcinica***	[NCBI: YP_117375]	448	232	118	51	47	6.32	-0.109
At1	*Arabidopsis thaliana*	[NCBI: NP_568547]	481	219	148	54	60	8.81	-0.039

Actinomycetes are highlighted in bold type.

The structural model of the protein tDGAT was aligned with its previously studied homolog Ma2 [8] through the software Pymol [34] (S3A Fig). Both proteins had identical predicted 3D structures and shared conserved motifs. Taking into account that the hydrophobicity of the environment surrounding the enzymes of this family has been suggested to significantly influence the activity and/or substrate specificity of WS/DGAT proteins [14,35], we compared the vacuum electrostatics prediction of both proteins (S3B Fig). The amphiphilic trait seems to be more pronounced in tDGAT than in Ma2, in spite of the higher average hydrophobicity observed in Table 1.

### Heterologous expression of tDGAT gives rise to rapid TAG accumulation in *Escherichia coli*

**Fig 2:**
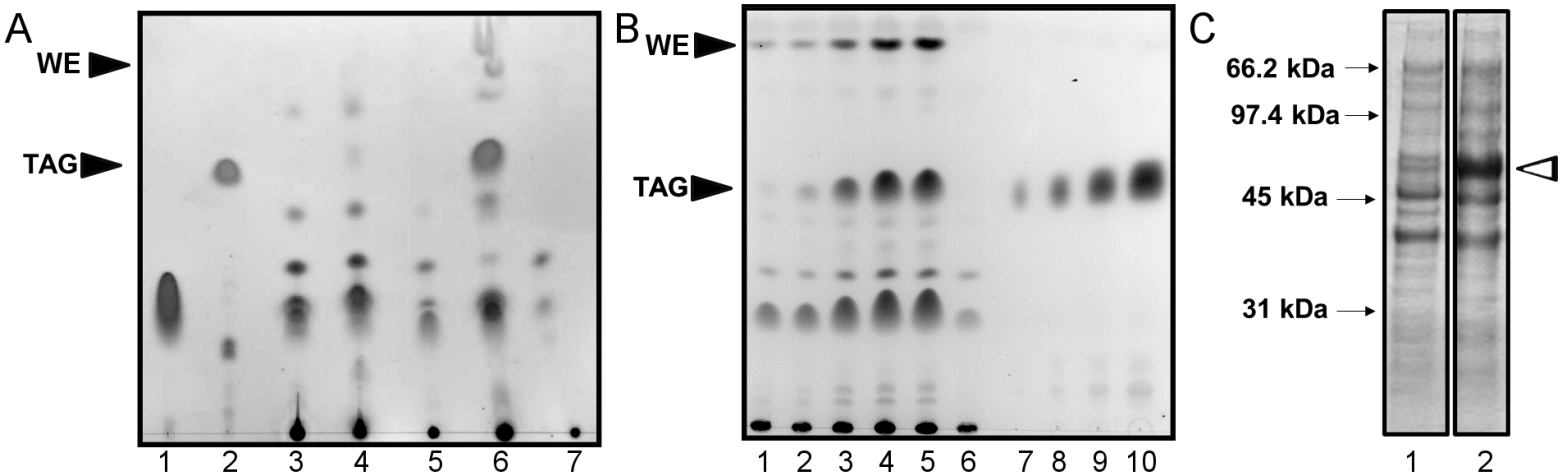
Detection of TAG production in the *E*. *coli* C41 (DE3) strain carrying the enzyme tDGAT and correlation with its expression. (A) TLC showing the analysis of the lipid fractions extracted from cultures of *E*. *coli* C41 (DE3) with the plasmid constructions pET29c::*Ma1* (lane 3), pET29c::*Ma2* (lane 4), pET29c::*Ab* (lane 5), pET29c::*tDGAT* (lane 6) and the naked plasmid pET29c (lane 7). Oleic acid (lane 1) and trioleoylglycerol (lane 2) were loaded as control standards. Positions of TAGs and WEs are marked by black arrows. (B) TLC plate showing TAG production in the engineered strain *E*. *coli* C41 (pET29c::*tDGAT*) along time after IPTG induction. Lipid extractions from 6.5 mg of dry biomass collected 0, 30, 60, 90 and 120 minutes after induction with 100 mM IPTG at OD_600nm_ = 0.5 were loaded in lanes 1–5, respectively. A similar lipid extraction from wild type *E*. *coli* was loaded in lane 6. Different amounts of purified commercial TAGs (4.5; 18; 36 and 72 μg) were loaded in lanes 7–10 in order to estimate the TAG production by densitometric assay. The black arrows point to the migration distance of the TAGs and WEs. (C) SDS-PAGE electrophoretic gel of whole cell lysates of *E*. *coli* (pET29c::*tDGAT*) collected after induction periods of 0 and 90 minutes (lanes 1 and 2). Molecular masses (in kilodaltons) are indicated at the left. The band indicated by the white arrowhead corresponds to the protein tDGAT.

Due to the high GC content and different codon usage of *T*. *curvata* the DNA sequence was codon-optimized for the expression of tDGAT in the gamma-proteobacteria *E*. *coli*. Subsequently, the optimized gene coding for tDGAT was cloned into a pET expression vector. The same procedure was employed for the other WS/DGATs tested (Methods). Thus, *E*. *coli* C41 cells were transformed with pET-derived plasmids containing DGAT genes from *M*. *hydrocarbonoclasticus* (*Ma1* or *Ma2*), *Alcanivorax borkumensis* (*Ab*) or *T*. *curvata* (*tDGAT*) and the profile of the accumulated neutral lipids was analyzed by thing layer chromatography (TLC) as described in Methods. Strikingly, the production of TAGs in *E*. *coli* was mainly observed in those cells expressing tDGAT (Fig 2A). A slight TAG production is also observed in cells expressing Ma2.

Although several reports can be found in the literature about TAG production in recombinant *E*. *coli* strains [12,18–22], little attention has been paid to culture times required for TAG production through these processes. Analysis of neutral lipid fractions of recombinant bacteria at different times after the induction of the expression of tDGAT showed the capacity of the engineered strain of producing TAGs in a very short period of time (Fig 2B). Densitometry analysis of TLC plates shows the ability of the engineered strain for accumulating 32.9% of its neutral lipids as TAGs just 90 minutes after induction, which translates into 1.25% of TAG/ dry biomass (w/w), or 4.8 mg TAG/ L. Simultaneously, the expression of tDGAT also gave rise to the production and accumulation of wax esters. Fig 2B shows the parallel accumulation of TAGs and WE with time after induction. The accumulation of TAGs and WEs in the recombinant *E*. *coli* strain was directly related to the expression of tDGAT (see S4 Fig). Strikingly, 90 minutes of induction are enough for a considerable production of the enzyme tDGAT (see Fig 2C).

One of the main interests in using *E*. *coli* for TAG production is their wide substrate range. Thus, *in vivo* functionality of tDGAT was also tested during the growth on glucose, gluconate, fructose, xylose, lactose and glycerol substrates. As observed in S5A Fig, similar TAG accumulation was obtained independently of the substrate chosen. Different temperatures (15–37°C) were also tested. Despite some differences in the rate of growth, TAG accumulation was detected to be similar in lipid extracts from cultures grown at 25, 30 or 37°C. However, TAG was not detected in cultures grown at 15°C (S5B Fig). To express tDGAT in *E*. *coli* strains not expressing the T7 phage RNA polymerase, the gene was cloned under a promoter regulated by arabinose (Methods). This construction was also shown to accumulate TAG when introduced into *E*. *coli* BW27783 (S5C Fig). TAG accumulation was slightly lower in this context possibly due to the differences in the promoter strength. Besides, in naturally oleogenic microorganisms like actinomycetes neutral lipid accumulation strongly depends on the nitrogen concentration, a stressing condition widely reported to enhance TAG accumulation [13]. However, TAG production in *E*. *coli* strains expressing tDGAT was observed to be independent of nitrogen availability in the growing media (S5D Fig).

### tDGAT is associated with the lipids accumulated in recombinant *E*. *coli* cells

While other bacterial WS/DGATs were easily purified and had *in vitro* WS and DGAT activity [7,8,36], tDGAT showed tendency to aggregate and *in vitro* activity could not be detected by spectrophotometric assays using Ellman’s reagent. This could be related to the particular GRAVY and pI values previously observed, indicating a different behavior of the thermophilic enzyme compared to other acyltransferases.

**Fig 3:**
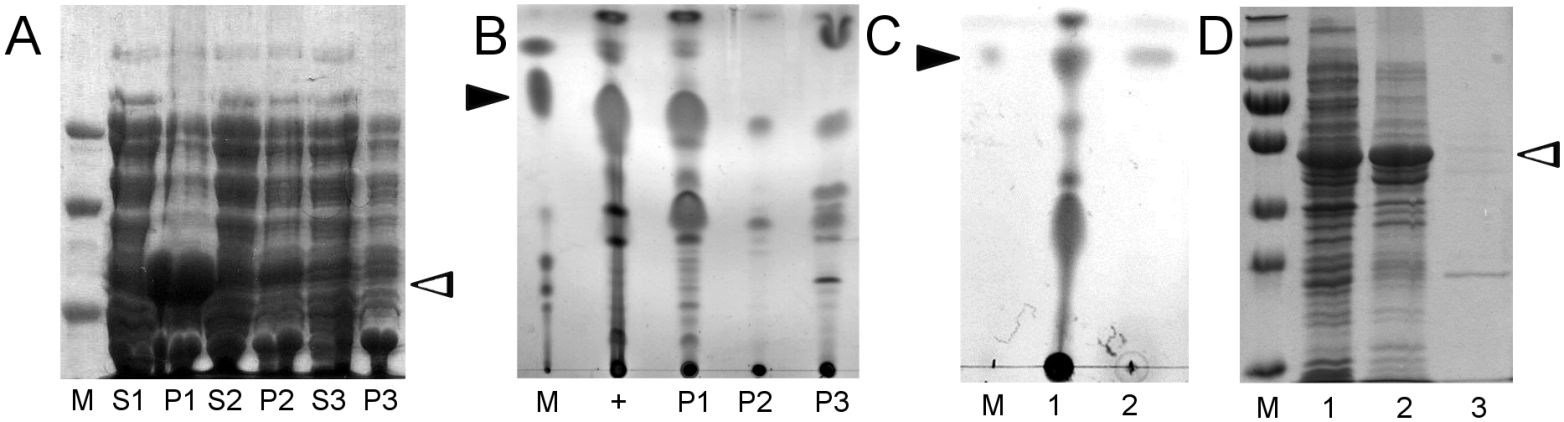
Ultracentrifugation experiments reveal tDGAT remains associated to the neutral lipids accumulated by the recombinant bacteria. **A and B, cell lysate of *E*. *coli* C41 (DE3) cells expressing tDGAT were sonicated and separated into phases by centrifugation. Samples from 1 to 3 correspond to consecutive centrifugations of the same sample at 2,000 g, 10,000 g and 55,000 g respectively**. (A) Electrophoretic 8% SDS-PAGE gel analysis of the supernatants (S1-S3) and pellets (P1-P3) of the different centrifugation fractions. tDGAT forming inclusion bodies falls at 2,000g. Lane M: Low Range SDS-PAGE Molecular Weight Standards (BioRad). (B) Thin layer chromatography (TLC) of lipid fractions extracted from 50 ml cultures expressing tDGAT. Lane +: Full pellet centrifuged at 55,000g. Lanes 1–4: Centrifugation pellets from P1 to P3. C and D, lipid bodies purification by sucrose gradient. (C) TLC of lipid fractions extracted from a cell lysate of *E*. *coli* C41 (DE3) cells expressing tDGAT: Lane M, triolein; lane 1, pellet obtained after centrifugation at 3,000 g and lane 2, the supernantant of the sucrose gradient centrifugation at 180,000 g. (D) Electrophoretic 12% SDS-PAGE gel analysis of the total *E*. *coli* C41 (DE3) cell lysate (1), proteins in the pellet obtained at 3,000 g (2) and proteins in the supernantant of the sucrose gradient centrifugation (3). Lane M shows the PageRuler Plus Prestained Protein Ladder (ThermoFisher). Position of TAGs are marked with black arrows in the TLC images. White arrowheads correspond to the protein tDGAT.

The direct relation between tDGAT overexpression and TAG production in tDGAT-expressing *E*. *coli* cells led us to check whether the protein itself is protecting TAGs from degradation. Analysis of the different cell fractions was performed by sonication and ultracentrifugation at different speeds to detect if tDGAT and neutral lipids were associated. In Fig 3A and 3B, SDS-PAGE and analytic TLC analyses of fractions from *E*. *coli* C41 (pET29c::*tDGAT*) cultures are shown. tDGAT remained in the pellet after ultracentrifugation at 2,000 g, while other WS/DGAT enzymes such as Ma1, Ma2 or Ab are known to be in the soluble cellular fraction [8,28,36]. Moreover, this fraction obtained at 2,000 g contained most of the neutral lipids. We have also isolated lipid bodies by sucrose gradient as described by Ding et al (2012) [37]. As observed in Fig 3C and 3D, only a small amount of TAGs is obtained from the top of the sucrose gradient while this fraction does not contain tDGAT. Hence, this experiment revealed that most of the lipids accumulated in the recombinant strain are somehow associated to the acyltransferase responsible for its biosynthesis.

### Optimization of fluorescence techniques for the detection of *in situ* TAG accumulation in *E*. *coli*

**Fig 4:**
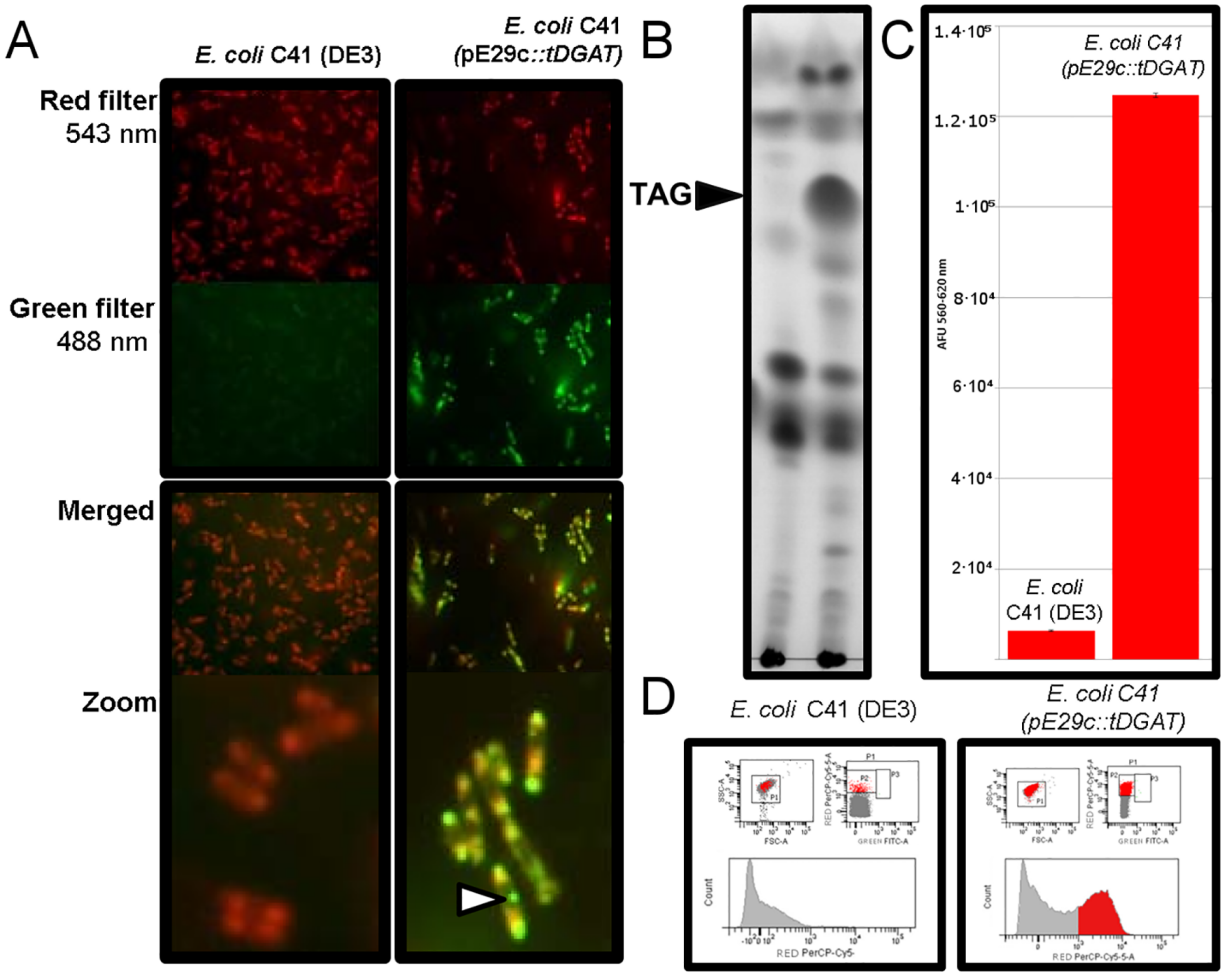
Correspondence between fluorescence and TAG production in Nile Red -stained cells. (A) Fluorescence microscopy images corresponding to the Nile Red -stained strains *E*. *coli* C41 (DE3) (left) and *E*. *coli* C41 (pE29c::*tDGAT*) (right) acquired with different wavelengths: red filter, 543 nm; green filter, 488 nm. Merged images of both fluorescence scans acquired and zoomed areas with fewer bacteria are displayed. The white arrow points to a lipid inclusion in the engineered tDGAT-expressing *E*. *coli* C41 (DE3) strain. (B) TLC plate showing the neutral lipid profile of the lipid extracts from the cultures analysed in the fluorescence experiments. Black arrow points to the TAGs. (C) Bar charts showing red (620 nm) average fluorescence spectroscopy measurements from both stained cultures. Error bars correspond to three experiments. AFU: Arbitrary Fluorescence Units. (D) Images of cytometry analysis of *E*. *coli* C41 (DE3) (left) and *E*. *coli* C41 (pE29c::*tDGAT*) (right) stained with Nile Red 20 h after induction of tDGAT expression with IPTG. An increase higher than 30% of the events were detected as fluorescent in the recombinant strain accumulating TAGs.

Detection methods implying lipid extraction are time consuming. In order to facilitate a rapid monitoring of the accumulation of neutral lipids in living *E*. *coli* cells, the activity of tDGAT was checked through different techniques based on fluorescence measurements *in vivo*. The selective lipophilic stain Nile Red binds to neutral lipids and emits intense fluorescent light when surrounded by hydrophobic environments [38]. Thus, we analysed by laser scanning microscopy the fluorescence produced by tDGAT recombinant cells in the presence of Nile Red. Nile red is known to specifically emit green fluorescence when associated with cytoplasmic lipid droplets [39]. Fig 4A shows how green fluorescence significantly increases in tDGAT recombinant cells in comparison with the respective wild types, concentrated specifically in spots likely to be lipid aggregates.

Fluorescence emitted by the Nile Red-stained recombinant *E*. *coli* C41 (pET29c::*tDGAT*) strain and its control was also measured by fluorescence spectrometry (Fig 4C) and flow cytometry (Fig 4D), as described in Methods. Fluorescence spectrometry shows that *E*. *coli* C41 (pET29c::*tDGAT*) treated with Nile Red emitted 1.2 x10^5^ AFU while the emission of the control *E*. *coli* C41 cells was 6.4 x10^3^ AFU. When equally treated cells were evaluated by flow cytometry, we observed that there is a shift in the cells detected as fluorescent at 695nm when excited with 488 nm laser. Among 30,000 cellular events analysed from each culture, 1.1% were fluorescent in the control bacteria while the percentage of fluorescent cells rose to 34.5% in *E*. *coli* C41 (pET29c::*tDGAT*). Thus, both methods show an increment higher than 30% in the detected fluorescence, which roughly matches the percentage of TAGs estimated by other analysis such as TLC densitometry (Fig 4B). This direct relation between fluorescence and TAG accumulation in both methods could be useful for the development of efficient assays to detect TAG-producing bacterial strains.

### The FA profile in *E*. *coli* changes towards MUFA enrichment upon tDGAT expression

Since the properties of the commercial products derived from SCO depend on the chemical characteristics of these lipids, characterization of the neutral lipids produced in bacteria expressing tDGAT is important. Different analytic techniques such as Nuclear Magnetic Resonance (NMR), Gas Chromatography (GC) and Ultra Pressure Liquid Chromatography/Mass Spectrometry (UPLC/MS) were employed to evaluate the alteration of the bacterial lipid profile in response to the heterologous expression the enzyme tDGAT.

**Fig 5:**
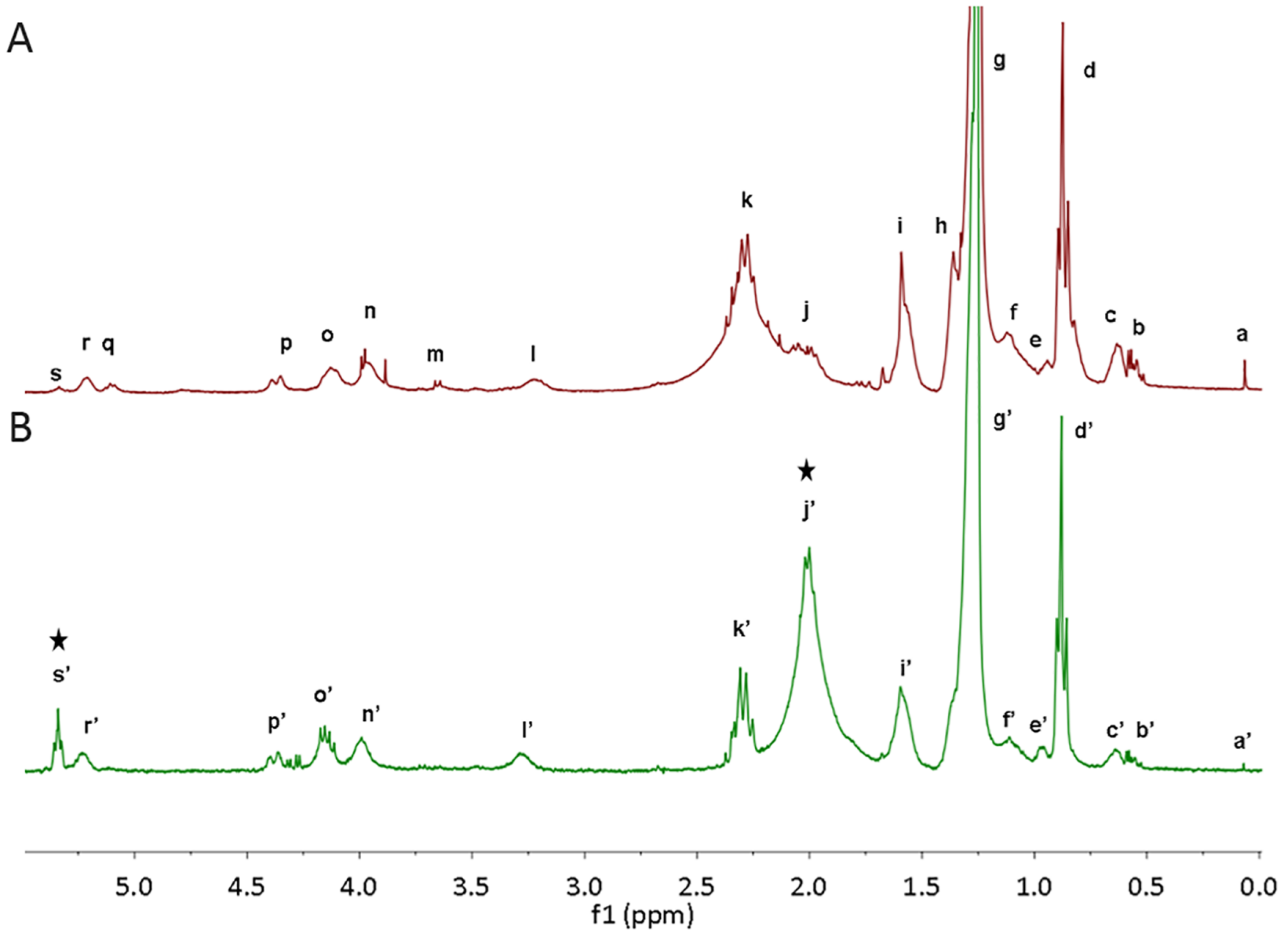
^1^H NMR spectra (CDCl_3_, 300.19 MHz) from lipid mixtures show an increase in the proportion of double bonds. Comparison of the 0.0–5.5 ppm regions of the ^1^H NMR spectra from the whole lipid extract of engineered *E*. *coli* C41 (DE3) cells expressing the tDGAT (B, low green spectrum) and the whole lipid extract of the same bacteria without any heterologous protein (A, upper red spectrum). Peaks are named from right to left and attributions are detailed in S1 Table. Black arrows point to the peaks indicating an increment in the proportion of double bonds in the recombinant strain. For more clarity, the spectrum is zoomed and only the region where all signals appear is shown. The only signal outside this region is the CDCl_3_ solvent residual peak (7.26 ppm, data not shown).

Firstly, we acquired Proton Nuclear Magnetic Resonance (^1^H NMR) of whole lipid extracts from 37°C-cultures of both *E*. *coli* C41 (DE3) expressing tDGAT and WT *E*. *coli* C41 (DE3). In Fig 5 we can observe that the most significant differences were the signals at 5.29 ppm and at 2.01 ppm. Variations in the abundance of certain species were calculated assuming that the perfect triplet at 0.88 ppm is due only to -CH_3_ terminal methyl protons (peak d and d’), and therefore normalizing the integrals of these signals as multiples of 3. The identification of the majority of the signal peaks was performed according to the literature [40–42]. This first highlighted signal (peaks s' and s), typical of olefinic protons, increased 22-fold when tDGAT was expressed (green spectrum), over the lipid extraction of the WT bacteria (red spectrum). Simultaneously, the signal at 2.01 ppm, typical of allylic protons, was also enhanced in the sample corresponding to *E*. *coli* expressing tDGAT (peak j’) where it increased 6-fold with respect to the WT bacteria (peak j). On the other hand, there was a 2-fold decrease in the signal at 2.29 ppm (peak k) in the samples from the bacteria expressing the tDGAT. According to the literature [40,41], this peak corresponds either to methylene protons adjacent to a carboxylic group or to H-2 protons of acyl moieties in acylglyceric or ester compounds. The observed differences suggest an accumulation of compounds with double bonds related to the activity of the protein tDGAT.

**Fig 6:**
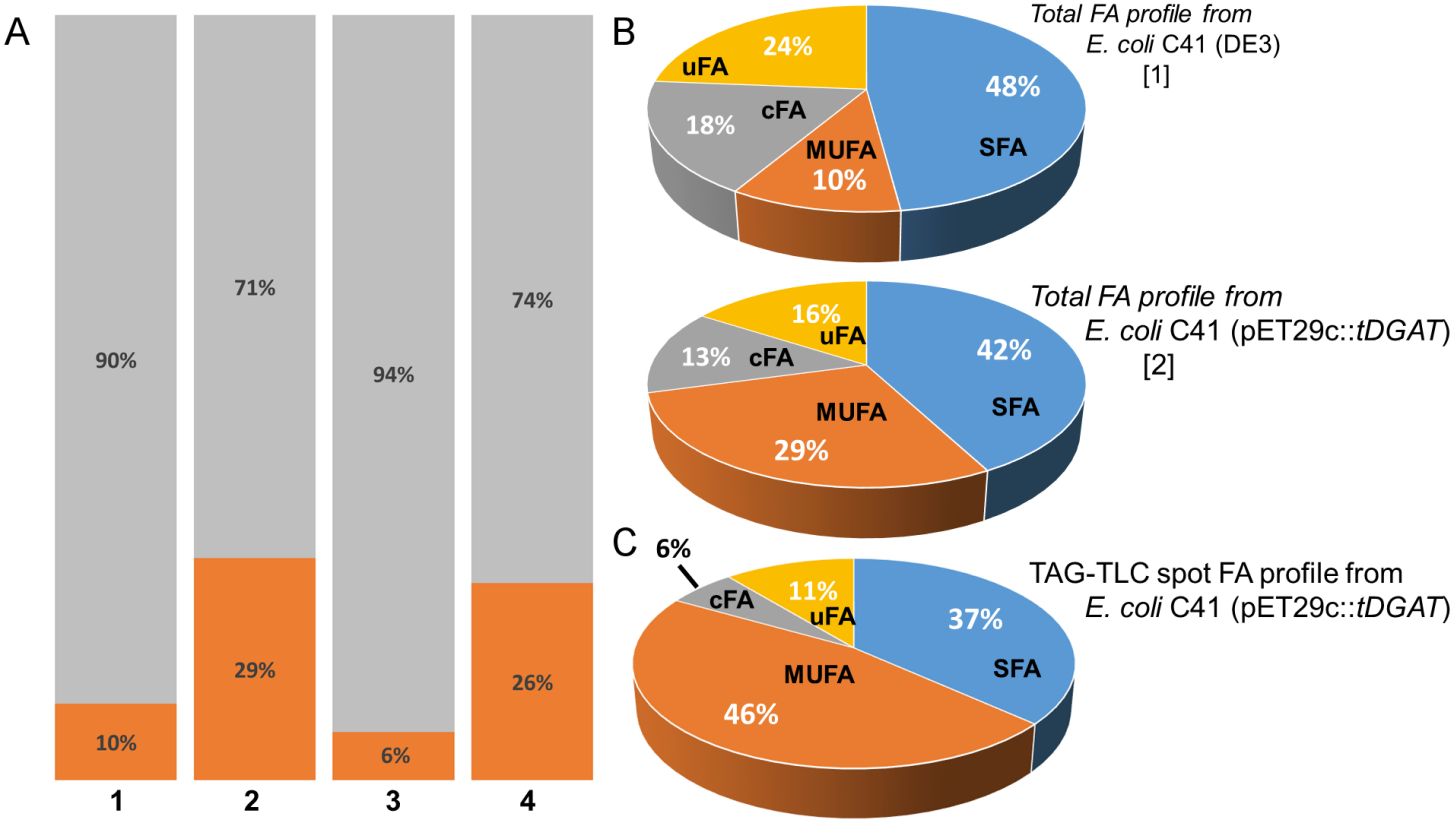
FA profile in *E*. *coli* cells expressing tDGAT. (A) Bar graphs showing the MUFA percentage (dark orange) among the whole FA pool in diverse *E*. *coli* strains [1: WT *E*. *coli* C41 (DE3); 2: *E*. *coli C41* (pET29c::*tDGAT*); 3: WT *E*. *coli* BW27783; 4: *E*. *coli* BW27783 (pBAD33::*tDGAT*)] (B) Circular graphs showing a more detailed comparison among the whole lipid extractions from *E*. *coli* C41 (DE3) [1] and *E*. *coli* C41 (pET29c::*tDGAT*) [2] with the TAG-TLC spot from *E*. *coli* C41 (pET29c::*tDGAT*) (C). MUFA are presented in dark orange, saturated FA in blue, confirmed cyclopropyl FA in grey and unidentified FA (inferred from literature) in yellow.

Further analysis to identify the nature of these double bonds was carried out by GC of total lipid extracts and isolated TAGs of *E*. *coli* C41 (DE3) strains expressing the protein tDGAT and their corresponding controls. As observed in Table 2, *E*. *coli* C41 (DE3) under our experimental conditions mainly contains palmitic acid (C16:0, 30.64%) and the cyclopropane FA methylene hexadecanoic acid (C17:0cycle, 17.75%). Interestingly, the analysis revealed a bias towards the monounsaturated fatty acids (MUFAs) palmitoleic (C16:1n7) and *cis*-vaccenic (C18:1n7) acids in the recombinant strain *E*. *coli* C41(pET29c::*tDGAT)* respect to the proportions of these FA occurring in the WT strain. This change also occurred in BW27783 expressing tDGAT under the pBAD expression system (Fig 6A). While MUFA proportion in both C41(DE3) and BW27783 WT *E*. *coli* strains was below 11% of the total FA, at least 25% of MUFA are detected in strains expressing the enzyme tDGAT. This suggests that a relevant part of the double bonds observed by ^1^H NMR were related to the FA profile.

**Table 2:** Summary of GC results.

Retention time (min)	FA name	Lipid number	Whole lipid *E*. *coli* C41	Whole lipid*E*. *coli* C41 (pET29c::*tDGAT*)	TAG*E*. *coli* C41 (pET29c::*tDGAT*)
7.981	Lauric	C12:0	6.14%	3.75%	0.87%
12.120	Miristic	C14:0	11.15%	6.90%	5.34%
17.096	Palmitic	C16:0	30.64%	31.58%	30.45%
17.871	Palmitoleic	C16:1n7	2.39%	8.11%	20.01%
20.402	C17:0 cycle	C17:0	17.75%	13.39%	5.77%
23.112	*cis* vaccenic	C18:1n7	8.22%	20.38%	26.58%
23.870	-	-	9.16%	6.76%	0.68%
25.586	-	-	8.20%	3.72%	2.39%

Average percentage of the main FAs occurring in each sample, calculated from three independent experiments.

Once we observed changes in the total FA profile in tDGAT-expressing *E*. *coli* strains, we decided to analyzed the FA composition of the TAG itself. For this analysis, TAG fractions of tDGAT-expressing *E*. *coli* cultures were purified through preparative TLC or dry column chromatography (DCC). The fraction corresponding to the TAGs in *E*. *coli* C41 (pET29c::*tDGAT*) was also analyzed by GC to identify the FA composition of these TAGs. TAGs accumulated by these bacteria were constituted by FA naturally occurring in the wild type strain (Fig 6C). Precisely, these neutral lipid molecules were mainly formed by the two MUFA palmitoleic and *cis* vaccenic acids together with the saturated palmitic acid (C16:0) (Table 2). While an increase from 10 to 29% in MUFA in the total FA profile was observed upon tDGAT-overexpression in *E*. *coli* C41 (DE3) (Fig 6B), this type of FAs constitutes up to 46% of the FA in TAGs of the *tDGAT-*expressing *E*. *coli* C41 strain (Fig 6C). This result highlights the increment in the MUFA proportion especially accumulated in the TAG compounds.

The same TAG fraction from *E*. *coli* C41 (pET29c::*tDGAT*) was analyzed through ^1^H NMR. The detailed comparison between the experimental spectrum of commercial tributirin and the fraction purified from the WT *E*. *coli* C41 (DE3) expressing tDGAT is shown in S7 Fig. According to the chemical shifts of the different peaks, its multiplicity and the literature data [40,43], signals were assigned to the most probable set of protons and attributed to the probable functional group (S2 Table). The protons of the glycerol backbone are responsible for the multiplet at 5.26 ppm and the doublet of doublets at 4.14 and 4.29 ppm. The triplet at 5.35 indicates some double bond present in the purified molecule. The rest of the signals are due to other protons in the acyl moieties. The peak at 1.57 ppm is probably the sum of two signals that overlap: the H-3 protons of acyl moieties in TAGs and residual molecules of H_2_O in the sample. The absence of peaks around 0.98 ppm confirms the absence of ω-3 acyl moieties. The number of protons was attributed to each signal in accordance to the integration of the peaks and the functional groups assignment [42]. This result confirmed the relevant content in MUFA previously seen in the TAG compounds accumulated by the tDGAT-expressing strain, suggesting a predominant ratio of one double bond (i.e. 2 olefinic protons) per TAG. Moreover, as expected, spectra acquired from different purified fractions from both recombinant and wild type strain showed that profiles typical of TAG compounds were only detected among the samples from engineered bacteria.

TAGs purified from the recombinant *E*. *coli* C41 (pET29c::*tDGAT*) were further analyzed by UPLC/MS. A variety of TAG molecular species was observed to occur in bacteria expressing tDGAT (S8A Fig). All these purified TAGs were constituted by FA naturally present in wild type *E*. *coli*, mainly C16:0, C16:1 and C18:1 as previously seen through other techniques (S8B Fig). The predominant TAGs found were C49:1 (8%) C50:1 (11%), C51:1 (16%), C52:0 (13%) and C52:1 (17%), most of them formed by one MUFA and two SFA. In fact, the proportion of TAGs constituted by 1 MUFA and 2 SFAs reached 66% while only 22% contained 3 SFAs and 12% contained 2 MUFAs and 1 SFA. These data correlates well with the high MUFA proportion found among TAG acyl chains and with the predominant ratio of 1 MUFA per TAG estimated from ^1^H NMR analysis.

## Discussion

In the present study, *E*. *coli* was genetically engineered to produce and accumulate TAGs by introducing the enzyme WS/DGAT from the thermophilic actinomycete *T*. *curvata*. The predicted 3D structure of the protein resulted to be a monomer composed of two domains connected by a helical linker (as occurs in other acyltransferases). A multiple sequence alignment of tDGAT with representative members of the WS/DGAT family signaled the presence of the key conserved motifs of these enzymes. The mismatch in the alignment of the amino acid residues corresponding to part of the helical loop in tDGAT seemed to be different than the linker in other WS/DGAT proteins. That is likely to be due to a low conservation in this region among the members of the family rather than a significant characteristic of tDGAT. Besides, the comparison of the biochemical properties of every amino acid sequence of the alignment points to tDGAT as the only enzyme among them with a hydrophobic average charge. According to the theoretical pI, tDGAT would be negatively charged in an environment with a neutral pH, like the rest of the WS/DGAT proteins from microorganisms accumulating mainly TAGs as storage compound, the oleogenic actinomycetes (Table 1). Nevertheless, the disposition of the hydrophobic residues in the structurally aligned protein models suggests that the amphiphilic trait seems to be more pronounced in tDGAT (S2B Fig). This might be related with the poor solubility of the tDGAT observed through purification and colorimetric activity assays. Furthermore, all the engineered *E*. *coli* strains tested in this work expressed the heterologous WS/DGAT proteins [8], but only the one with the protein from a thermophilic organism expressed a functional enzyme gave rise to a rapid production of TAGs in living cells. According to Coleman et al. [25] the challenge of maintaining the structure of their proteins in spite of increased thermal disorder faced by organisms evolved at high temperatures results in differences in residue utilization and overall structure of the enzymes. Thus, the origin of the thermophilic protein tDGAT might explain its different behavior in a mesophilic microorganism like *E*. *coli*, compared to the activity of other proteins belonging to the same prokaryotic family when overexpressed in this model bacteria. Moreover, it has been suggested that the activity and/or substrate specificity of WS/DGAT proteins could be influenced by the hydrophobicity of the environment it is exposed to [14,35]. This fact can be explained by the amphiphilic character of the enzyme, which sharply contrasts with the highly hydrophobic eukaryotic WS [24].

Up to now, low yields of other lipid compounds like jojoba oil-like WEs, fatty acid butyl esters (FABEs),FAEEs and fatty-acid-derived hydrocarbons [15,16,44] had been achieved through the heterologous expression of the WS/DGAT enzyme of *A*. *baylyi* ADP1. Besides, recombinant TAG production has been achieved through the heterologous expression of a WS/DGAT in *E*. *coli* strains with further genetic modifications [12,17–22], whereas TAG production in a non-modified *E*. *coli* had not been achieved. Remarkably, the *in situ* activity of the tDGAT protein not only occurred in an extremely short period of time, but was also independent of other factors such as the growth conditions, the induction system or the employment of different *E*. *coli* strains. This versatility is relevant for a potential industrial application of this recombinant organism.

Ultracentrifugation revealed an association between the protein tDGAT and the neutral lipids produced. This connection is probably related both with the hydrophobicity of the amino acid sequence calculated by bioinformatics tools and the high amphipathic character of tDGAT. This way, the union protein-lipid droplets is likely to form insoluble lipoprotein inclusion bodies. Hence, this attachment suggests an important bottleneck for the increase of bacterial TAG production yield in the bacteria *E*. *coli* through this strategy. Further studies and modifications are required to improve this process. Besides, the correspondence observed between fluorescence and accumulation of neutral lipids observed when treating cultures with lipophilic stains such as Nile Red provides an important tool to detect genetic and metabolic changes modifying neutral lipid generation. This data set stablishes the basis for the development of a high throughput analysis method permit efficient optimization of the oil production.

Comparative analysis of the neutral lipids in *E*. *coli* C41 (pET29c::*tDGAT*) and the corresponding WT strain revealed a significant accumulation of lipid compounds with double bonds related to the accumulation of TAGs. Importantly, these double bonds appeared to be mainly related to the FA chains in the lipid fraction, since MUFA increased in 2.9-fold among the total FA in *E*. *coli* C41 (DE3) upon overexpression of tDGAT. Moreover, the analysis of the purified TAG fraction from *E*. *coli* C41 (pET29c::*tDGAT*) showed that 46% of the FA in these compounds were MUFA. According to our results, the majority of the TAGs produced by the engineered bacteria were constituted by one MUFA and two SFA. This suggests that the increment in MUFAs in the total FA profile observed in the recombinant strain is probably due to the accumulation of these unsaturated fatty acids in the synthesized TAGs. The FA profile in *E*. *coli* varies with the strain and growth conditions. For instance, it is known that MUFA proportion increases while culture temperature decreases [45]. Besides, increments in the MUFA cellular content have been previously described in literature in response to the heterologous expression of different proteins [46,47]

Among WS/DGAT enzymes, Lin and co-workers reported in 2013 [20] a 3–3.7- fold increase in the MUFA fraction for different *E*. *coli* strains expressing WS/DGAT enzymes from different microorganisms over FA composition of the wild type strain. Besides, Röttig and colleagues [22] also described a bias toward MUFA in an engineered *E*. *coli* strain overexpressing several proteins (including a bacterial WS/DGAT) and producing TAGs. Hence, this effect is not particular of tDGAT. Likewise, Rucker and co-workers [12] observed that almost half of the FA in the TAG produced by another engineered *E*. *coli* strain were MUFA. However, the explanation for this metabolic response is yet unclear. Our hypothesis relies either in the chemical protection FA acquire when becoming part of the TAG compounds (from spontaneous degradation such as β-oxidation) or in a higher selectivity of tDGAT for MUFA, which could produce an increase in the biosynthesis of these FA. Both possible interpretations would explain the increase in MUFA in the whole bacterial FA pool as well as the high MUFA content of the TAGs produced by the recombinant strain. The similarity of this bias in the FA content could also be related to the important function of TAG compounds in the cells as “sink” for unnecessary FA [1,48]. The bifunctional enzymes belonging to the bacterial WS/DGAT family have been reported to have an incredibly broad substrate range [49]. Nevertheless, this lack of specificity contrast with the bias towards MUFA observed both in the total FA profile of the engineered bacteria and in the TAG compounds accumulated. A deeper understanding on the catalytic mechanism of the enzyme tDGAT may lead to a huge improvement of the biotechnological applications of this protein.

TAG employed for the production of biofuels are currently of vegetal origin, which explain its high content in PUFA. That negatively impacts the properties of the biofuel. On the other hand, TAGs only formed by SFA are not appropriate either due to its poor performance at low temperatures [9,50,51]. Biodiesel have been said to be preferably originated from oils enriched in MUFAs [10,20]. Thus, a highly efficient production of oil enriched in MUFA would be the doorway towards the production of a better biodiesel [9,50]. The particular composition of the TAGs produced by the recombinant *E*. *coli* strain suggests this is an attractive way to develop an alternative process for obtaining TAGs for biodiesel production. Further improvement on the industrial conditions that favor the production of TAGs enriched in MUFA in this engineered bacteria might lead to yields that permits the establishment of alternative models for biofuel production.

## Conclusions

The remarkable high TAG producing activity shown *in vivo* by the thermophilic WS/DGAT enzyme reported in this work makes it a promising asset on the way to achieve a sustainable production system for this valuable material. This process needs to be improved by overcoming the TAG-production limit observed in the recombinant bacteria due to the binding of the tDGAT enzyme to the neutral lipids produced (probably related to the hydrophobicity of the protein). In this respect, we stablished the basis for the evaluation of the lipid content in different species. Furthermore, the particularity and importance of the enrichment in MUFA caused by this protein inspire further investigation in the characterization of the enzymes of this family or the development of novel processes with important biotechnological applications such as biodiesel production.

## Methods

### Structural modelling

The tDGAT 3D structure was predicted by homology modelling using the Phyre server (24; version 2.0 [http://www.sbg.bio.ic.ac.uk/phyre2/html/page.cgi?id=index]). The crystal structure of surfactin A synthetase c (SRFA-C), a nonribosomal peptide synthetase termination module [PDB: 2VSQ] was used as template. An image of the resulting 3D model structurally aligned with the 3D model of Ma2 was generated using the program Pymol (version 1.3 [http://pymol.org/dsc/])(28). The electrostatic surface representation was calculated for both models.

### Sequence analysis software and public gene expression data sets

All protein and nucleic acid sequences were obtained from the public databases at the National Center for Biotechnology Information (http://www.ncbi.nlm.nih.gov). Protein database searches were performed using BLASTP [27], available on the NCBI web site. Multiple sequence alignments were made using the software program T-coffee [52] (http://www.ebi.ac.uk/Tools/msa/tcoffee/*)* and plotted using the program ESPript 3 [53] (http://espript.ibcp.fr/ESPript/ESPript/). Further parameters obtained with the amino acid sequence were calculated through the Expasy web tools (http://www.expasy.org).

### Strains and culture conditions

The *Escherichia coli* strain DH5alpha [54] was employed for cloning procedures. *E*. *coli* C41 (DE3) [55] for protein expression and *in vivo* experiments. *E*. *coli* BW27783 [56] was used for *in vivo* assays.

Liquid cultures were prepared in flasks containing ¼ volume LB medium (Pronadisa, Spain) supplemented with either kanamycin sulphate (Sigma Aldrich, St. Louis, MO) at a final concentration of 50 μg / ml or chloramphenicol (Sigma Aldrich, St. Louis, MO) at 25 μg / ml and incubated at 37°C and 180 rpm. For solid media culture LA was employed (LB medium supplemented with 1.5% (w/v) agar; Pronadisa, Spain).

For testing the carbon source, minimal medium (M9 salts, MgSO_4_ 2mM and CaCl_2_ 0.1 mM with D-glucose, sodium gluconate, fructose, xylose, lactose or glycerol 40 g/l) was used.

### Protein expression and induction of lipid accumulation

Cultures of *E*. *coli* C41 (DE3) or BW27783 with the corresponding DNA constructions were grown to an OD_600nm_ value of 0.5–0.6 and the expression of the enzyme was induced with either isopropyl β-D-1-thiogalactopyranoside (IPTG) 100 uM or 0.05% arabinose.

### DNA manipulation and plasmid construction

A DNA sequence including the codon-optimized WS/DGAT gene from the oleogenic actinomycete *Thermomonospora curvata* was designed for *Escherichia coli* by Life Technologies (Waltham, MA). Native DNA sequences from the rest of the microorganisms tested in this work were employed due to its higher similarity to the *E*. *coli* GC content. All DNA fragments were amplified by chain polymerase reaction (PCR) with oligonucleotides purchased from Sigma-Genosys (Sigma-Aldrich, St. Louis, MO) and the high fidelity DNA polymerase Vent (BioLabs, UK). It was then inserted into the plasmid vector through standard DNA technology procedures employing DH5alpha cells as host strain. *Nde*I and *Xho*I restriction sites were used to introduce the DNA fragments into a pET Bacterial Expression Vector (pET29c; Novagen, Madison, WI). A ribosome binding site was inserted together with the gene of interest into an arabinose inducible and glucose repressible pBAD plasmid (pBAD33 [57], carrying a pACYC184 origin of replication and a gene conferring chloramphenicol resistance) by employing the restriction enzymes *Kpn*I and *Hind*III. Restriction enzymes, Shrimp Alkaline Phosphatase and T4 DNA ligase, were purchased from Thermo Scientific (Waltham, MA). The polymerase Biotaq (Bioline, London, UK) was used for PCR comprobation of genetic constructions.

DNA was analyzed through agarose gel electrophoresis (0.7–1% p/v agarose in TBE buffer) employing Hyperladder I (Biolabs, UK) as molecular weight marker, 0.05 mg/ml SYBR Safe (Invitrogene, Life Technologies, Waltham, MA) for staining, a horizontal running system (BioRad, Hercules, CA) and a Quantity One software (BioRad, Hercules, CA) to visualize images taken by a Gel Doc 2000 UV system. Plasmid DNA isolation was performed by the commercial kit QIAprep Spin Miniprep (QIAGEN, Germany). PCR products were purified with the kit GenElute PCR Clean-Up (Sigma-Aldrich, St. Louis, MO) or extracted from electrophoretic gels by employing the kit GenElute Gel Extraction (Sigma-Aldrich, St. Louis, MO). DNA concentration was determined at 260 nm with a Nano-Drop ND-1000 spectrophotometer (Thermo Scientific, Waltham, MA).

Microdialized (Millipore GS, 0.05 μm pore size) DNA ligations were transformed into electroporation competent DH5α, BW27783 or C41 (DE3) *E*. *coli* cells previously prepared according to standard molecular biology protocols [58] by using 0.2 cm Gene Pulser cuvettes (BioRad, Hercules, CA) in a MicroPulserTM electroporator (BioRad, Hercules, CA). *E*. *coli* C41 (DE3) with plasmid constructions pET29c::*Ma1*, pET29c::*Ma2*, pET29c::*Ab* and pET29c::*tDGAT* and *E*. *coli* BW27783 cells with plasmid construction pBAD33::*tDGAT* were employed in subsequent experiments.

### Protein analysis

#### Protein purification

*E*. *coli* C41(DE3) with plasmid constructions pET29c::*Ma1*, pET29c::*Ma2*, pET29c::*Ab* and pET29c::*tDGAT* were grown in 2 l flasks containing 1 l LB supplemented with 50 ug/ml kanamycin sulfate and induced with 1 mM IPTG when the OD_600nm_ was approximately 0.6. Cell cultures were collected 3 hours later by centrifuging in a JA10 rotor (Beckman Coulter, Brea, CA) at 4,400 g for 15 minutes a 4°C and stored frozen at -20°C until analyzed. The supernatants were discarded and the pellets were resuspended in buffer A (NaCl 1 M, TrisHCl 50 mM pH 7.5 and PMSF 100 μl), sonicated and clarified by ultracentrifugation at 100,000 g for 15 minutes at 4°C. Supernatants were loaded onto a Níckel HisTrap HP column (GE Healthcare, 5 ml) previously equilibrated with buffer A. The His-tagged bound proteins were eluted by a gradient of concentration of imidazole (0-300mM; Sigma, St. Louis, MO). In order to improve the purification, the eluted protein was concentrated using Amicon Ultra 30k Centrifugal filters (Millipore, Ireland) and loaded into a size-exclusion chromatography Superdex 75 GL10_30 column (Amersham Pharmacia Biotech, UK) previously equilibrated with buffer B (NaCl 150 mM TrisHCl 50 mM pH 7.5 1 mM EDTA). Purified proteins were stored at -80°C in glycerol 20% for further experiments.

#### Protein electrophoresis

Protein electrophoresis was carried out in 10% polyacrylamide SDS gels. Samples were prepared by adding 150 μL of loading buffer (Tris-Cl 50 mM pH 6.8; SDS 4% (w/v); bromophenol blue 0.02% (w/v); glycerol 4% (v/v) and 100 mM β-mercaptoethanol) to 1 mL-culture-pellets centrifuged at 2,000 g, 10,000 g or 55,000 g Coomassie Brilliant Blue R-250 (Merck, Germany) was used for staining and a solution of methanol/acetic acid/deionized water (4:1:5, v/v/v) for destaining. Low Range Protein Marker (BioRad, Hercules, CA) was used as molecular weight marker and electrophoresis was developed for 45 min at 170 V and 300 mA.

### Lipid extraction

To evaluate lipid production cells were collected at different growing times by centrifugation at 3,100 g for 10 minutes. Lipids were extracted using an adapted solvent-based method described by Hara et al. [59]. A solution of hexane / 2-isopropanol (3:2, v/v) (both purchased from Sigma-Aldrich, St. Louis, MO) was added to every cell pellet previously thawed at room temperature. After vigorously vortexing, tubes were left for 3 hours with shaking. Then they were centrifuged for 7 min at 12,000 g and 8°C, the upper organic phase was collected and placed into new 2 mL tube and the solvent was evaporated in a rotational vacuum concentrator (Christ RVC2-18, Germany) at 40°C.

#### Ultracentrifugation analysis of tDGAT-contaning lipoprotein complexes

An overnight 50 ml culture of *E*. *coli* C41 (DE3) cells that expressed the tDGAT protein for 3 hours were pelleted, resuspended in buffer (Tris 50mM pH 7.5; NaCl 150mM) and sonicated. The broken cells were subjected to a series centrifugation cycles to pellet and separate different subcellular parts. In this way, the cell fractions where tDGAT protein and neutral lipids remain associated were identified. For this purpose, the sample was subjected to consecutive centrifugations at 2,000 g., 10,000 g. and 55,000 g., harvesting the supernatant in each centrifugation step for using it in the next one. In each step a sample of the supernatant and a sample of the pellet were analyzed by SDS-PAGE and lipid extraction followed by TLC.

Lipid bodies were isolated essentially as described by Ding et al.2012 [37]. Briefly, an overnight 1 L culture of *E*. *coli* C41 (DE3) cells that expressed the tDGAT *protein* was harvested by centrifugation at 3,000 g for 10 min, and resuspended in 80 ml of buffer A (25 mM tricine pH 7.8 and 250 mM sucrose). After 20 min incubation on ice, cells were lysed by a constant cell disruptor (constant systems) treatment twice at 40,000 psi. The sample was then centrifuged at 3,000 g for 10 min to collect cell debris and inclusion bodies. Supernatant (around 80 ml) was loaded into ultracentrifuge tubes with 20 ml buffer B (20 mM HEPES pH 7.4, 100 mM KCl, 2 mM MgCl_2_) on top, and was centrifuged at 180,000 g for 1 h at 4°C. The fraction on top of the sucrose gradient was collected and the lipids were extracted and analysed by TLC.

### Lipid analysis

#### Analytical Thin Layer Chromatography (TLC)

A solution consisting of hexane/diethyl ether acid/acetic acid (80:20:1, v/v/v) was employed as mobile phase and a 20 × 20 × 0.02 cm Polygram Sílica Gel (Macherey-Nagel, Germany) as stationary phase to carry out the separation of lipid species. Every lipid extraction was resuspended in 14 μl of hexane / 2-isopropanol (3:2) and loaded by 2 μl-drops onto the Silica Gel. Standard markers for lipid characterization (Fatty Acid Alkyl Esters, FAEEs; Triacylglycerides, TAGs and Free Fatty Acids, FFAs; Sigma, St. Louis, MO) were also loaded. Staining of the samples was performed using iodine vapour. A densitometric analysis of scanned images of the TLC plates was carried out through the software Fiji (ImageJ) as previously described [60].

#### Fatty Acid Methyl Esters conversion and gas chromatography

Spots corresponding to TAGs from both the recombinant strain *E*. *coli* C41 (DE3) carrying the DNA construction pET29c::*tDGAT* and the wild type strain were scrapped out from the TLC plate with a scalpel and put into 2 mL tubes. The resulting mixtures of lipids-silica gel as well as the cell pellet of whole cultures of *E*. *coli* C41 (pET29c::*tDGAT)* and the wild type strain *E*. *coli* C41 (DE3), were converted into Fatty Acid Methyl Esters (FAMEs) through a protocol adapted from Miller and Berger [61]. Saponification was performed by adding 1.25 ml solution 15% NaOH (w/v) in methanol/deionized water (1:1, v/v) to each sample in glass tubes (Quality PYREX^®^, 16x100 glass test tubes with screw cap), the mixture was incubated at 100°C for 5 minutes, vortexed and incubated at 100°C for 25 more minutes. Methylation was carried out by adding 2.5 ml HCl 3N in methanol/water (13:11, v/v) and incubating at 80°C for exactly 10 minutes. Lipids were extracted with 1.25 ml of hexane/ ethylic eter (1:1, v/v) and shaking for 20 minutes. The 1 ml upper solution obtained after decanting was washed with a solution of 1.2% NaOH in deionized water, vortexed, briefly centrifuged for decanting and the 0.5 ml upper phase was transferred to a new vial.

The fatty acid profile was determined through the analysis of the FAMEs with a gas chromatograph (Model 7890A, Agilent, Santa Clara, CA) at the company Biomar Microbial Technologies (León, Spain). Analytes were separated on a column model DB-23 (60 m, 0.25 mm, 0.25 μm, Agilent, Santa Clara, CA) using a temperature program of 130°C for 1 min followed by a temperature rise of 2.75°C per minute, 12 minutes at 215°C followed by a temperature ramp of 40°C per minute, 3 minutes at 230°C and finally 130°C for 1 min. Constant flow of helium as a carrier gas was set at 20 ml/min. The flow of H_2_ was 30 ml/min and N_2_ at 22.5 ml/min was employed as auxiliary gas. Sample sizes of 2 μL were injected. The FID detector was set at 280°C.

FA identification was carried out from linear-chain FA equivalents and through comparison to standards in retention time. FAs with retention times ranging from hexanoic acid (C6) to tetracosane acid (C24) were calculated according to Miller y Berger [61]. Standards were purchased from Sigma (St. Louis, MO).

#### Ultra Pressure Liquid Chromatography/Mass Spectrometry (UPLC/MS) analysis

The spots corresponding to the TAGs purified from TLC plates both from the recombinant strain *E*. *coli* C41 (DE3) carrying the DNA construction pET29c::*tDGAT* and from the wild type strain were further separated in a column Acquity UPLC BEH HSS T3 (100 × 2.1 mm, 1,7 μm p.s) and a Vanguard Pre-column (100 × 2.1 mm) employed through the equipment Acquity UPLC System (Waters, Milford, MA) with a sample manager and a binary solvent manager. Solvents employed were methanol/acetonitrile/2-isopropanol (3:3:4, v/v/v; solvent A) y acetonitrile/2-isopropanol (3:7, v/v; solvent B), both with 0.1% NH_4_OH (25%). Elution was carried out from a simple gradient with the following steps: initial, 100% A; 3 min, 100% A; 6 min, 98% A; 8 min, 98% A; 9.5 min, 95% A; 11 min, 95% A; 16 min, 100% A. Between every sample, methanol was injected twice to wash and avoid the carry over.

Mass spectrometry was performed in order to identify the lipid species present in every sample in a SYNAPT G2 HDMS (Waters, Milford, MA) equipment, with quadrupole time of flying (QToF) and electrospray ionization (ESI). The capillary was set at 0.8 kV; sampling cone, 15 V; temperature, 90°C; desolvation temperature, 280°C; cone gas, 40 L/h; and desolvation gas, 700 L/h. Data was acquired from 5 scans/s in the range 0–18 min and 100–1200 Da for low energy function and 100–900 for the high energy function (base pick fragmentation, collision energy 30 V), with positive ionization (ESI^+^). Mass signatures (m/z ratios) of parent and daughter fragment ions were determined experimentally under direct injection using glyceryl tripalmitate (16:0/16:0/16:0), glyceryl triestearate (18:0/18:0/18:0), glyceryl trioleate (18:1/18:1/18:1), glyceryl trilinolenate (18:3/18:3/18:3) and glyceryl trilinoleate (18:2/18:2/18:2) as representative standards purchased from Sigma (St. Louis, MO). The observed mass of the parent ion matched the mass of the triglyceride plus NH_4_^+^, a component of the mobile phase solvent. Both the lipid purification and the MS analysis were carried out in CDB (Valladolid, Spain).

#### Proton Nuclear Magnetic Resonance (^1^H NMR)

NMR analysis of mixtures of lipids or pure lipids were performed in a FOURIER-300 MHz spectrometer (B_0_ = 7.05 T, Bruker, Germany) at 23°C and 300.19 MHz using deuterated chloroform (CDCl_3_, Sigma-Aldrich, St. Louis, MO) as solvent. For all the pulse sequences, packages from Bruker were used. 700 μl of sample solutions containing 2–8 mg of purified lipids were placed in a 5 mm-diameter NMR tube (in such a way the length of the tube filled with the liquid sample occupied approximately 4.5 cm long). All spectra were recorded with sample rotation and the chemical shifts are expressed in δ scale (ppm), referenced to the residual signal of chloroform (7.26 ppm) [62]. Signals were characterized as singlet (s), doublet (d), doublet of doublets (dd), triplet (t) or multiplet (m). The software TopSpin was employed to acquire the spectra and all data were analysed thought the software MestreNova. The assignments were performed mainly according to [40–42,63]. The peaks corresponding to solvent non-deuterated (>1%) and trace impurities were identified following the method described in [64].

### Lipid purification

#### Preparative TLC plate

Samples were loaded onto 20 × 20 × 0.1 cm TLC plates and the analysis technique was developed as above explained. The spots corresponding to the lipids to be analysed were scrapped out from the silica-gel plate with a scalpel and put into 2 mL Eppendorf tubes together with 1 mL n-hexane/2-isopropanol (3:2, v/v). The samples were left for 3 hours with shaking, then centrifuged for 7 minutes at 12,000 g and 8°C and the upper phase was collected (in such a way no silica gel particles were picked) into new Eppendorf tubes. The samples were then concentrated in a rotational vacuum concentrator (Christ RVC2-18, Germany).

#### Dry-Column Flash Chromatography (DCC)

The laboratory glassware was chosen and the protocol defined as previously described [65] to purify different lipid fractions within a total extract. Analytical TLCs were performed to determine the composition of each sample obtained.

### Fluorescence staining

Samples of *E*. *coli* C41 (DE3) cells with/without the plasmid construction pET29c::*tDGAT* were prepared by incubation of the cultures previously grown without any staining molecule for 30 minutes at 4°C in a solution with 0.5 μg/ml of the lipophilic dye Nile Red (stock solution prepared at 0.5 mg/ml in DMSO, Sigma, St. Louis, MO).

#### Fluorescence microscopy imaging

Nile Red treated *E*. *coli* C41 cell cultures were collected by centrifugation at 4°C and 16,000 g and resuspended in methanol at -20°C for 2 min. Pellets were collected again by centrifugation, resuspended in a volume of phosphate buffered saline (PBS) and spread onto microscope pads with Vectashield (Vector Laboratories, Burlingame, CA). Samples were examined under the laser scanning microscope LSM 510 (Zeiss, Germany) with a 63X oil inmersion lens (1,4 NA). All settings of the microscope were identical for every image acquisition. Images were acquired with emission filters of 488 nm and 543 nm.

#### Flow cytometric analysis

The percentage of fluorescent cells in the different cultures was detected by a BD FACSCanto^™^II flow cytometer (BD Biosciences, San Jose, CA) using FACSDiva software (BD Biosciences, San Jose, CA). Samples were excited with a 488 nm laser and emission was detected at 695nm. 30,000 events were acquired for every sample.

#### Fluorescence spectroscopy analysis

A fluorimeter (Victor3, Perkin Elmer, Santa Clara, CA) was employed to measure the arbitrary fluorescence units emitted by every sample. 96-well microtiter plates (Deltalab, Barcelona, Spain) were used to perform the different assays. An excitation laser wavelenght of 560 nm was used to stimulate samples and a filter of 620 nm was employed to detect the fluorescence emitted. A stabilized lamp energy of 30,000 units and emission aperture “above” and “small” were set for all measurements.

## Supporting information

S1 Fig Multiple sequence alignment of representative enzymes belonging to the WS/DGAT family. The coding sequences for the proteins were obtained from annotated protein data bases and are listed in Table 1. Identical residues are shown in white on a red background, while similar residues are shown in red. The secondary structure elements of the modelled tDGAT protein are shown above the alignment. Secondary structure representation is coloured blue for the N-terminal domain, red for the C-terminal domain, and green for the connecting helices. The active site motif HHxxxDG [33] is remarked with black stars.
(TIF)

S2 Fig Docking model of tDGAT and two common substrates. (A) PalmitoylCoA as acyl donor (in orange) and a DAG molecule as acyl acceptor (in purple) are modelled with tDGAT catalytic motif HHxxxDG (highlighted in yellow sticks) on the predicted three dimensional structure. (B) Zoomed view of the PalmitoylCoA molecule entering the central tunnel on a predicted surface of the protein tDGAT. The catalytic motif is highlighted with a black star and the motif II is also labelled in both images. The model is colored blue for the N-terminal domain, red for the C-terminal domain, and green for the connecting helices.
(TIF)

S3 Fig Structural comparison of tDGAT with the Ma2 protein. (A) Structural alignment of the 3D prediction analysis of tDGAT (purple) and Ma2 (white). Residues corresponding to the active site motif characteristic of the WS/DGAT family are highlighted in orange and marked with a black star. The other main conserved motifs also responsible for the activity of these proteins are colored in green and labeled as mI and mII [8]. (B) Predicted electrostatic surface of both proteins (upside: tDGAT; downside: Ma2) overlapped onto the ribbon representation of the skeleton. Both sides of the proteins are shown. The active site is marked with a black star. Zoomed views of the catalytic motif are also shown in both enzymes.
(TIF)

S4 Fig Correlation of TAG production in the *E*. *coli* C41 (DE3) strain carrying the enzyme tDGAT and its expression. (A) TLC showing the analysis of the lipid fractions extracted from cultures of E. coli C41 (DE3) with the plasmid construction pET29c:: *tDGAT* collected 3, 6 and 24 hours (lanes 4, 5 and 6, respectively) after induction and the naked plasmid pET29c (lane 3). Oleic acid (lane 1) and trioleoylglycerol (lane 2) were loaded as control standards. The black arrowhead shows the migration distance of the TAGs. (B) SDS-PAGE electrophoretic gel of whole cell lysates of *E*. *coli* (pET29c::*tDGAT*) collected after induction periods of 0, 3, 6 and 24 hours (lanes 2–5). Protein Marker was loaded in lane 1. The band indicated by the white arrowhead corresponds to the protein tDGAT according to its predicted molecular weight.
(TIF)

S5 Fig Lipid analysis of tDGAT-expressing *E*. *coli* strain over diverse conditions. (A) TLC plates showing lipid extracts of cultures of *E*. *coli* C41 (pET29c::*tDGAT*) grown on defined media with different carbon sources at the same concentration in every case (glucose in lane 2; gluconate in lane 3; fructose in lane 4; xylose in lane 5; lactose in lane 6; glycerol in lane 7). Lane 1 corresponds to the lipid extract from the wild type *E*. *coli* C41 (DE3) grown in rich medium (LB broth). (B) Lipid extracts of cultures of *E*. *coli* C41 (pET29c::*tDGAT*) grown in rich medium (LB Broth) at different temperatures (15°C in lane 1; 25°C in lane 2; 30°C in lane 3; 37°C in lane 4). (C) Lipid extracts from different strains carrying the tDGAT enzyme under diverse expression systems: *E*. *coli* BW27783 (pBAD33::*tDGAT*) in lane 2 and *E*.*coli* C41 (pET29c::*tDGAT*) in lane 3. A parallel control extract from *E*.*coli* C41 (DE3) without the heterologous gen was loaded in lane 1. (D) TLC plates showing TAG production in tDGAT-expressing *E*. *coli* cells grown under diverse conditions of nitrogen availability and carbon source. Lipid extractions loaded onto lanes 1, 3, 5 correspond to cultures made on minimal media supplemented with 1 g/l NH_4_Cl, while those on lanes 2, 4, 6 come from cultures grown on minimal media with 0.05 g/l NH_4_Cl. All cultures contain 1% of carbon source: glucose (1, 2), gluconate (3, 4) or arabinose (5, 6). The black arrows point to the migration distance of the TAGs.
(TIF)

S6 Fig GC chromatograms showing the shift in the FA profile due to the functionality of the heterologous enzyme in living cells. Gas chromatograms obtained from the analysis of the FAMEs from the whole cell culture of both the wild type *E*. *coli* C41 (DE3) (A) and the recombinant strain *E*. *coli* C41 (pET29c::*tDGAT*) (B) as well as from the extracted TLC spots corresponding to TAGs (C) of the engineered strain. The main FAs occurring in different percentages in the samples are labeled: tetradecanoic acid (C14:0), palmitic acid (C16:0), palmitoleic acid (c16:1n7), heptadecanoic acid (C17:0cicle), *cis* vaccenic acid (C18:1n7).
(TIF)

S7 Fig Characterization of the predominant TAGs produced by *E*. *coli* (pET29c::*tDGAT*). Comparison of the 0.0–5.5 ppm region of the ^1^H NMR spectra (CDCl_3_, 300.19 MHz) from the commercial TAG tributirin (4:0/4:0/4:0) (purple upper spectrum) and a lipid compound purified from the recombinant microorganism *E*. *coli* C41 (pET29c::*tDGAT*) (green low spectrum). For clarity, only the zoomed region between 5 and 5.5 ppm is shown, since this region where most of signals appear. The only signal outside this region is the CDCl_3_ solvent residual peak (7.26 ppm, data not shown).
(TIF)

S8 Fig UPLC/MS analysis of TAGs accumulated in *E*. *coli* C41 (pET29c::*tDGAT*). (A) Extracted ion chromatogram (EIC) of the TAG TLC-spot of *E*. *coli* C41 (pET29c::*tDGAT*). (B) Main TAGs found in recombinant strain.
(TIF)

S1 Table Peak attributions of lipid extracts ^1^H NMR spectra. Chemical shift assignments of ^1^H NMR signals indicated in Fig 5. Abbreviations: s, singlet; d, doublet; t, triplet; m, multiplet.
(PDF)

S2 Table Assignments of the different peaks of the ^1^H NMR spectra of purified TAG compounds. Chemical shift attributions of ^1^H NMR signals indicated in S7 Fig. Abbreviations: s, singlet; d, doublet; dd, doublet of doublets; t, triplet; m, multiplet.
(PDF)

S1 File DNA sequence of the protein tDGAT codon-optimized for *E*. *coli*. (PDF)

## Abbreviations

^1^H NMR: proton nuclear magnetic resonance
DAG: diacylglycerol
FA: fatty acid
FAEEs: fatty acid ethyl esters
FAMEs: fatty acid methyl esters
GC: gas chromatography
MS: mass spectrometry
MUFA: mono unsaturated fatty acids
PUFAs: poly unsaturated fatty acids
TAG: triacylglycerol
TLC: thin layer chromatography
UPLC/MS: ultra-high pressure liquid chromatography/ mass spectrometry
WS/DGAT: wax ester synthase/ triacylglycerol:acylCoA acyltranferase
WT: wild type
