# Supplementary figures and images for: Heterologous expression of a thermophilic diacylglycerol acyltransferase triggers triglyceride accumulation in Escherichia coli
Source: PLoS One. 2017 Apr 27;12(4):e0176520. doi: 10.1371/journal.pone.0176520 (PMC5407786; doi:10.1371/journal.pone.0176520)

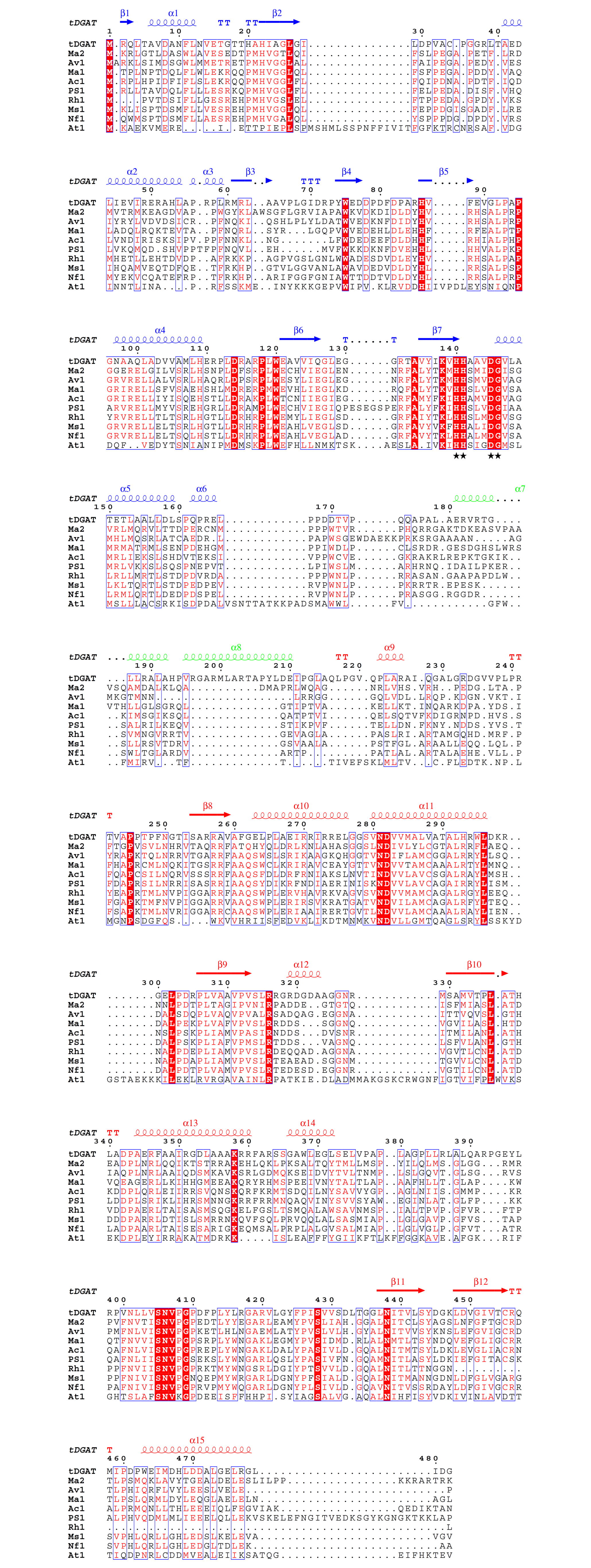

Supplement: S1 Fig — The coding sequences for the proteins were obtained from annotated protein data bases and are listed in Table 1. Identical residues are shown in white on a red background, while similar residues are shown in red. The secondary structure elements of the modelled tDGAT protein are shown above the alignment. Secondary structure representation is coloured blue for the N-terminal domain, red for the C-terminal domain, and green for the connecting helices. The active site motif HHxxxDG [33] is remarked with black stars. (TIF) [file pone.0176520.s001.tif]

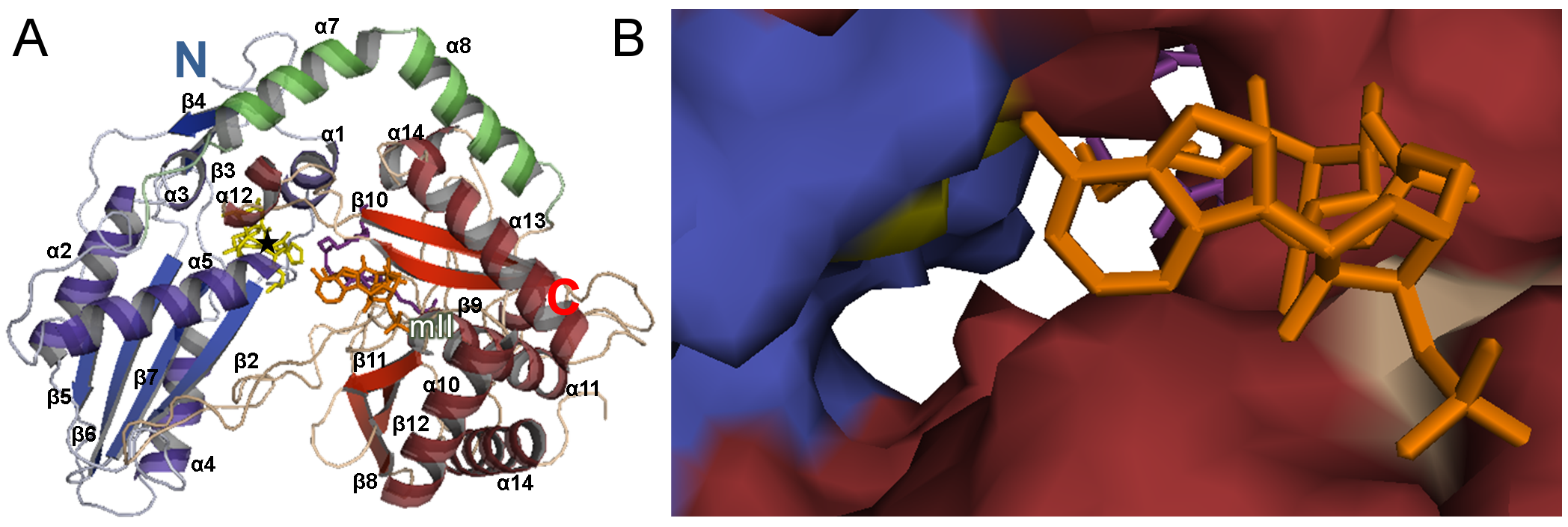

Supplement: S2 Fig — (A) PalmitoylCoA as acyl donor (in orange) and a DAG molecule as acyl acceptor (in purple) are modelled with tDGAT catalytic motif HHxxxDG (highlighted in yellow sticks) on the predicted three dimensional structure. (B) Zoomed view of the PalmitoylCoA molecule entering the central tunnel on a predicted surface of the protein tDGAT. The catalytic motif is highlighted with a black star and the motif II is also labelled in both images. The model is colored blue for the N-terminal domain, red for the C-terminal domain, and green for the connecting helices. (TIF) [file pone.0176520.s002.TIF]

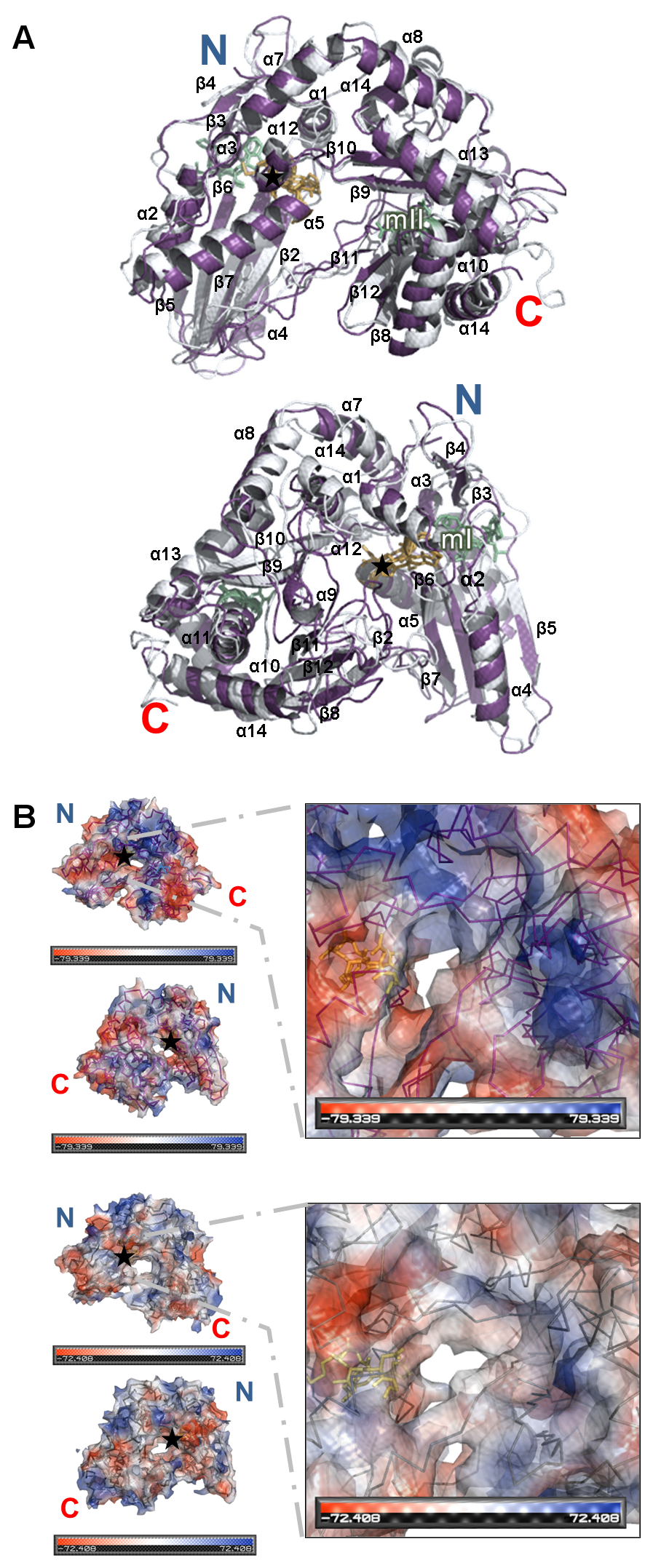

Supplement: S3 Fig — (A) Structural alignment of the 3D prediction analysis of tDGAT (purple) and Ma2 (white). Residues corresponding to the active site motif characteristic of the WS/DGAT family are highlighted in orange and marked with a black star. The other main conserved motifs also responsible for the activity of these proteins are colored in green and labeled as mI and mII [8]. (B) Predicted electrostatic surface of both proteins (upside: tDGAT; downside: Ma2) overlapped onto the ribbon representation of the skeleton. Both sides of the proteins are shown. The active site is marked with a black star. Zoomed views of the catalytic motif are also shown in both enzymes. (TIF) [file pone.0176520.s003.tif]

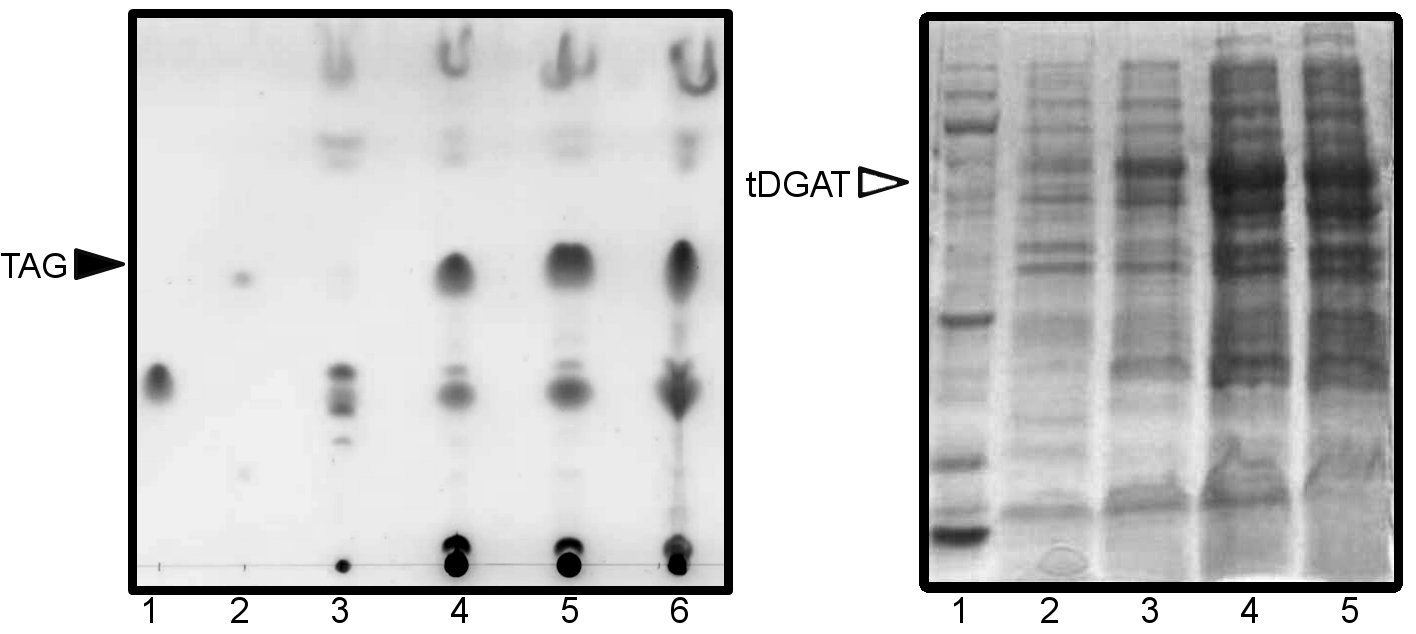

Supplement: S4 Fig — (A) TLC showing the analysis of the lipid fractions extracted from cultures of E. coli C41 (DE3) with the plasmid construction pET29c:: tDGAT collected 3, 6 and 24 hours (lanes 4, 5 and 6, respectively) after induction and the naked plasmid pET29c (lane 3). Oleic acid (lane 1) and trioleoylglycerol (lane 2) were loaded as control standards. The black arrowhead shows the migration distance of the TAGs. (B) SDS-PAGE electrophoretic gel of whole cell lysates of E. coli (pET29c::tDGAT) collected after induction periods of 0, 3, 6 and 24 hours (lanes 2–5). Protein Marker was loaded in lane 1. The band indicated by the white arrowhead corresponds to the protein tDGAT according to its predicted molecular weight. (TIF) [file pone.0176520.s004.tif]

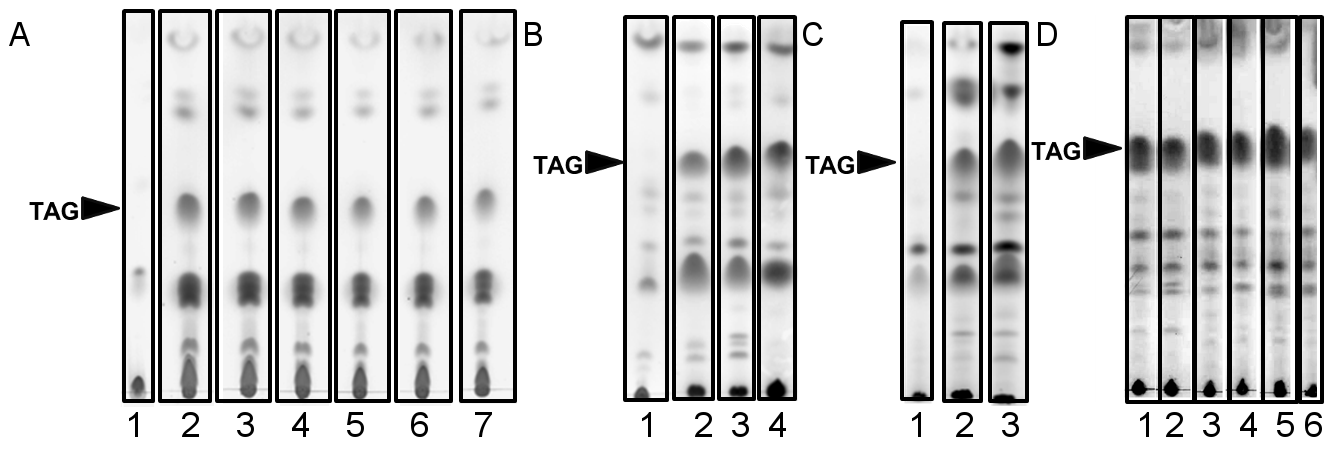

Supplement: S5 Fig — (A) TLC plates showing lipid extracts of cultures of E. coli C41 (pET29c::tDGAT) grown on defined media with different carbon sources at the same concentration in every case (glucose in lane 2; gluconate in lane 3; fructose in lane 4; xylose in lane 5; lactose in lane 6; glycerol in lane 7). Lane 1 corresponds to the lipid extract from the wild type E. coli C41 (DE3) grown in rich medium (LB broth). (B) Lipid extracts of cultures of E. coli C41 (pET29c::tDGAT) grown in rich medium (LB Broth) at different temperatures (15°C in lane 1; 25°C in lane 2; 30°C in lane 3; 37°C in lane 4). (C) Lipid extracts from different strains carrying the tDGAT enzyme under diverse expression systems: E. coli BW27783 (pBAD33::tDGAT) in lane 2 and E.coli C41 (pET29c::tDGAT) in lane 3. A parallel control extract from E.coli C41 (DE3) without the heterologous gen was loaded in lane 1. (D) TLC plates showing TAG production in tDGAT-expressing E. coli cells grown under diverse conditions of nitrogen availability and carbon source. Lipid extractions loaded onto lanes 1, 3, 5 correspond to cultures made on minimal media supplemented with 1 g/l NH4Cl, while those on lanes 2, 4, 6 come from cultures grown on minimal media with 0.05 g/l NH4Cl. All cultures contain 1% of carbon source: glucose (1, 2), gluconate (3, 4) or arabinose (5, 6). The black arrows point to the migration distance of the TAGs. (TIF) [file pone.0176520.s005.tif]

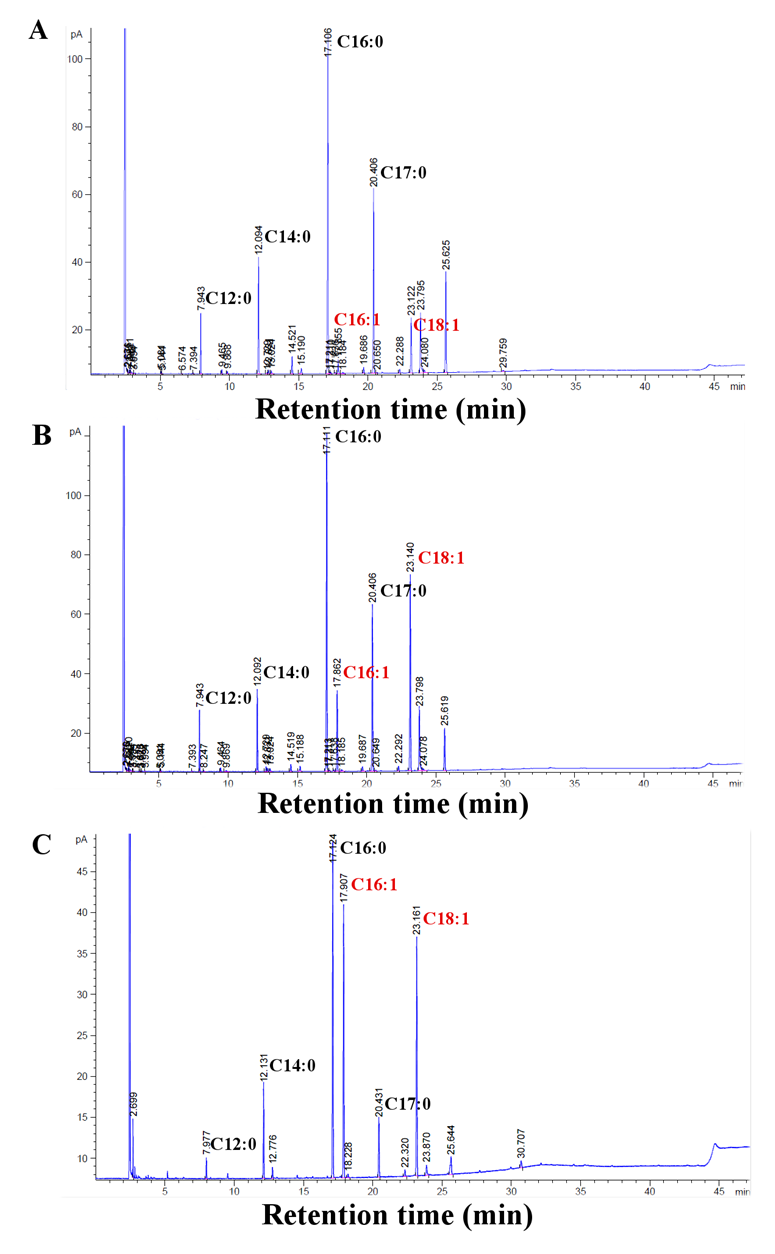

Supplement: S6 Fig — Gas chromatograms obtained from the analysis of the FAMEs from the whole cell culture of both the wild type E. coli C41 (DE3) (A) and the recombinant strain E. coli C41 (pET29c::tDGAT) (B) as well as from the extracted TLC spots corresponding to TAGs (C) of the engineered strain. The main FAs occurring in different percentages in the samples are labeled: tetradecanoic acid (C14:0), palmitic acid (C16:0), palmitoleic acid (c16:1n7), heptadecanoic acid (C17:0cicle), cis vaccenic acid (C18:1n7). (TIF) [file pone.0176520.s006.tif]

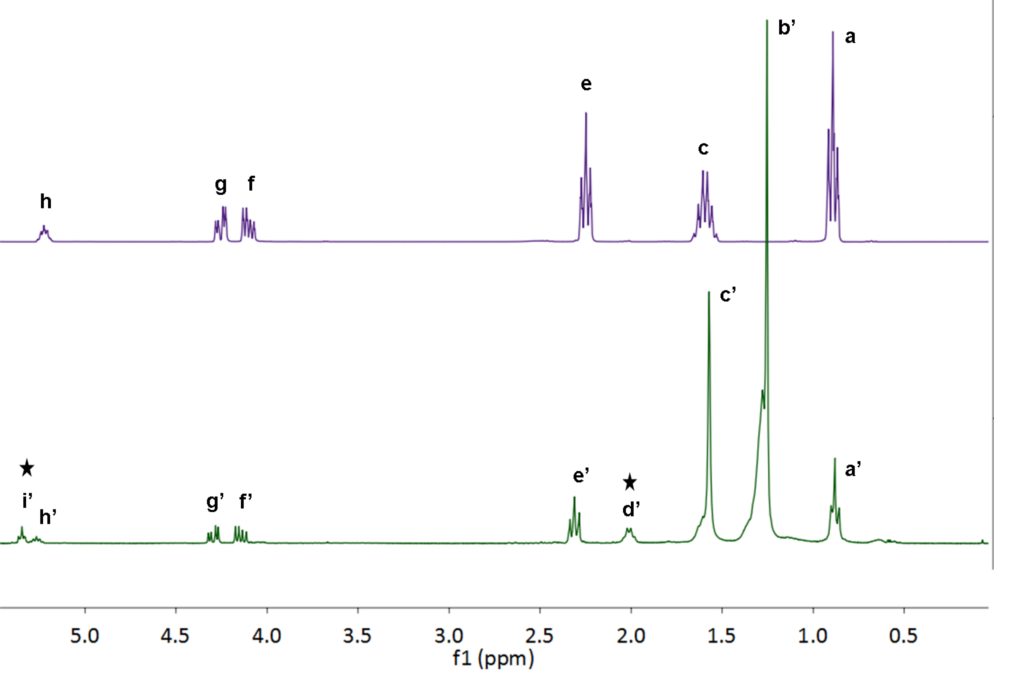

Supplement: S7 Fig — Comparison of the 0.0–5.5 ppm region of the 1H NMR spectra (CDCl3, 300.19 MHz) from the commercial TAG tributirin (4:0/4:0/4:0) (purple upper spectrum) and a lipid compound purified from the recombinant microorganism E. coli C41 (pET29c::tDGAT) (green low spectrum). For clarity, only the zoomed region between 5 and 5.5 ppm is shown, since this region where most of signals appear. The only signal outside this region is the CDCl3 solvent residual peak (7.26 ppm, data not shown). (TIF) [file pone.0176520.s007.tif]

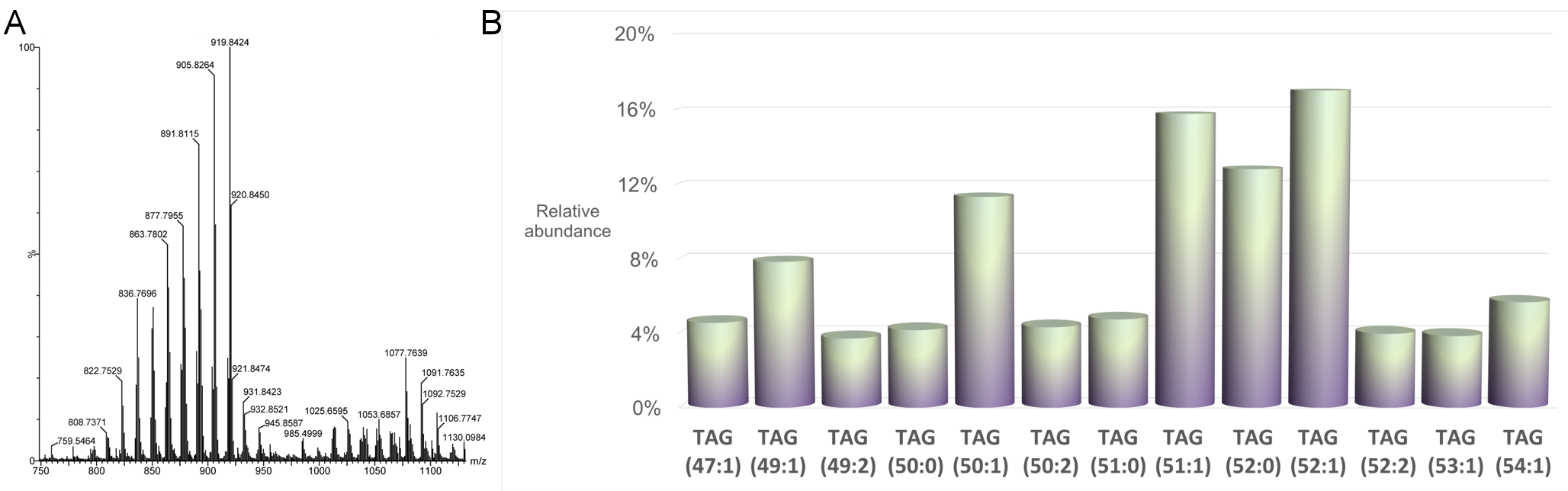

Supplement: S8 Fig — (A) Extracted ion chromatogram (EIC) of the TAG TLC-spot of E. coli C41 (pET29c::tDGAT). (B) Main TAGs found in recombinant strain. (TIF) [file pone.0176520.s008.tif]
