# Supplementary material for: Heterologous expression of a thermophilic diacylglycerol acyltransferase triggers triglyceride accumulation in Escherichia coli
Source: PLoS One. 2017 Apr 27;12(4):e0176520. doi: 10.1371/journal.pone.0176520 (PMC5407786; doi:10.1371/journal.pone.0176520)
Supplement: S1 Table — Chemical shift assignments of 1H NMR signals indicated in Fig 5. Abbreviations: s, singlet; d, doublet; t, triplet; m, multiplet. (PDF) [file pone.0176520.s009.pdf]

Table 1S – Peak attributions of lipid extracts ^1H NMR spectra.

Chemical shift assignments of ^1H NMR signals indicated in Figure 5. Abbreviations: s, singlet; d, doublet; t, triplet; m, multiplet.

Peak	Chemical shift, δ (ppm)	Multiplicity	Proton/ Functional group	Attribution	Reference
**a, a'**	0.07	s	$CH_3[Si(CH_3)_2O]_nSi(CH_3)_3$	Silicone grease: Polydimethylsiloxane (PDMS)	Gottlieb et al., 1997
**b, b'**	0.50-0.60	m			
**C, c'**	0.60-0.69	Broad signal			
**D, d'**	0.88	t	$-CH_3$	Terminal methyl protons in every acyl chain	Vlahov 1999
**E, e'**	0.92-0.99	Broad signal	$-CH_3$	^{13}C satellite of signal at 0.88 ppm	Alonso-Salces et al., 2012
**F, f'**	1.00-1.16	Broad signal			
**G, g'**	1.25		$-CH_2-$	Methylene envelop of various compounds	Vlahov 1999
**h**	1.36				

**i, i'**	1.59		-OCO-CH_2-C*H_2*-	H-3 protons of acyl moieties in acylglyceric or ester compounds	Vlahov 1999
**j, j'**	2.01	Broad signal	=CH-C*H_2*-	Allylic methylenes	Vlahov 1999
**k, k'**	2.29	m	i) HOOC-C*H_2*-	i) Methylene protons adjacent to a carboxylic group	Alonso-Salces et al., 2012
			ii) -CO-C*H_2*-	ii) H-2 protons of acyl moieties in acylglyceric or ester compounds	Vlahov 1999
**l, l'**	3.22/3.28/3.18	Broad signal			
**m**	3.65	d	-C*H_2*OH	Glyceryl group in DAGs	Alonso-Salces et al., 2012
**n, n'**	3.90-4.04	Broad signal			
**o, o'**	4.05-4.23	m	C*H*-OH	Glycerol group in DAGs	Almoselhy 2014
			-C*H_2*-OR	H-1/H-3 proton of glycerol group TAG	Vlahov 1999
**p, p'**	4.30-4.44	d	-C*H_2*-OR	H-1/H-3 proton of glycerol group TAG	Vlahov 1999
**q**	5.10	m			

**r, r'**	5.16-5.26	Broad signal	>C*H*-OR	H-2 proton in TAGs	Vlahov 1999
**s, s'**	5.29	t	=C*H*-	All UFA	Vlahov 1999
**t, t'**	7.26 (not shown)			$CDCl_3$ solvent residual peak	Gottlieb et al., 1997
