# Supplementary material for: Heterologous expression of a thermophilic diacylglycerol acyltransferase triggers triglyceride accumulation in Escherichia coli
Source: PLoS One. 2017 Apr 27;12(4):e0176520. doi: 10.1371/journal.pone.0176520 (PMC5407786; doi:10.1371/journal.pone.0176520)
Supplement: S2 Table — Chemical shift attributions of 1H NMR signals indicated in S7 Fig. Abbreviations: s, singlet; d, doublet; dd, doublet of doublets; t, triplet; m, multiplet. (PDF) [file pone.0176520.s010.pdf]

Table 2S. Assignments of the different peaks of the ^{1}H NMR spectra of purified TAG compounds.

Chemical shift attributions of ^{1}H NMR signals indicated in Figure S7. Abbreviations: s, singlet; d, doublet; dd, doublet of doublets; t, triplet; m, multiplet.

**Peak Name (agree with figure)**	**Chemical Shift, δ (ppm)**	**Peak multiplicity**	**Assigned number of H's**	**Proton-Functional group**
**a/a'**	0.89/0.88	t	9	-CH_3 → (terminal methyl protons of saturated and unsaturated chains)
**b'**	1.25	Broad signal	~70	-CH_2- →(Protons of methylene envelop)
**c/c'**	1.59/1.57	m	6	-COO-CH_2-CH_2- → (H-3 protons of acyl moieties in triacylglycerols)<br>H_2O → (residual molecules)
**d'**	2.01	m	4	=C- CH_2- → (allylic methylenes)
**e/e'**	2.25/2.31	t	6	COO- CH_2- → (H-2 protons of acyl moieties in triacylglycerols)
**f/f'**	4.10/4.14	dd	2	-CH_2-OR → (H-1 and H-3 protons of glycerol,<br>the assignments are interchangeable)
**g/g'**	4.25/4.29	dd	2	
**h/h'**	5.23/5.26	m	1	>CH-OR → (H-2 of the glycerol backbone)
**i'**	5.35	t	2	=CH– → (olefinic protons of unsaturated FA)
