# Supplementary material for: Heterologous expression of a thermophilic diacylglycerol acyltransferase triggers triglyceride accumulation in Escherichia coli
Source: PLoS One. 2017 Apr 27;12(4):e0176520. doi: 10.1371/journal.pone.0176520 (PMC5407786; doi:10.1371/journal.pone.0176520)
Supplement: S1 File — (PDF) [file pone.0176520.s011.pdf]

S1 File. DNA sequence of the protein tDGAT codon-optimized for *E. coli*

atgcgtcagt taacagcagt tgatgcaaat tttctgaatg ttgaaaccgg caccacccat

gcacatattg caggtctggg tattctggat ccggttgcat gtccgggtgg tcgtctgacc

gcagaagatc tgattgaagt tattcgtgaa cgtgcacatc tggcaccgcg tccgctgcgc

atgcgtctgg ctgcagttcc gctgggtatt gatcgtccgt attgggaaga tgatccggat

tttgatccgg cacgtcatgt ttttgaagtt ggtctgcctg caccgggtaa tgcagctcag

ctggcagatg ttgttgcaat gctgcatgaa cgtcctctgg atcgtgcacg tccgctgtgg

gaagcagttg ttattcaggg tctggaaggt ggtcgtaccg cagtttatat taaagttcat

catgcagccg ttgatggtgt tctggcaacc gaaaccctgg cagcactgct ggatctgagt

ccgcagcctc gtgaactgcc tccggacgat accgttccgc agcaggcacc ggcactggca

gaacgtgttc gtaccggtct gctgcgtgca ctggcacatc cggttcgtgg tgcacgtatg

ctggcacgta ccgcaccgta tctggatgaa attccgggtc tggcacagct gcctggtgtt

cagcctctgg cacgcgcaat tcagggtgca ctgggtcgtg atggtgttgt tccgctgcct

cgtaccgttg cacctccgac cccgtttaat ggcaccatta gcgcacgtcg tgcagttgca

tttggcgaac tgccgctggc agaaattcgt cgtattcgtc gcgaactggg tggtagcgtt

aatgatgttg ttatggcact ggttgcaacc gcactgcatc gttggctgga taaacgtggt

gaactgccgg atcgtccgct ggttgcagcc gttccggtta gcctgcgtcg tggccgtgat

ggtgatgcag ccggtggtaa tcgtatgagc gcaatggtta cacctctggc aacccatctg

gcagatccgg cagaacgttt tgcagcaatt cgtggtgatc tggcagcagc aaaacgtcgc

tttgcacgta gcagcggtgc atggctggaa ggtctgagcg aactggttcc ggcacctctg

gcaggtccgc tgctgcgtct ggcactgcag gcacgtccgg gtgaatatct gcgtccggtt

aatctgctgg ttagcaatgt tccgggtccg gattttccgc tgtatctgcg tggtgcccgt

gttctgggtt attttccgat tagcgttgtt agcgatctga ccggtggtct gaatattacc

gttctgagct atgatggcaa actggatgtt ggtattgtta cctgtcgtca gatgattccg

gatccgtggg aaattatgga tcatctggat gatgcactgg gtgaactgcg tggtctgatt

gatggt
